# Apoptotic Efficacy of Etomoxir in Human Acute Myeloid Leukemia Cells. Cooperation with Arsenic Trioxide and Glycolytic Inhibitors, and Regulation by Oxidative Stress and Protein Kinase Activities

**DOI:** 10.1371/journal.pone.0115250

**Published:** 2014-12-15

**Authors:** María Cristina Estañ, Eva Calviño, Susana Calvo, Beatriz Guillén-Guío, María del Carmen Boyano-Adánez, Elena de Blas, Eduardo Rial, Patricio Aller

**Affiliations:** 1 Centro de Investigaciones Biológicas (CIB), Consejo Superior de Investigaciones Científicas (CSIC), Ramiro de Maeztu 9, Madrid, Spain; 2 Departamento de Biología de Sistemas, Unidad de Bioquímica y Biología Molecular, Facultad de Medicina y Ciencias de la Salud, Universidad de Alcalá, Alcalá de Henares, Madrid, Spain; National Cheng Kung University, Taiwan

## Abstract

Fatty acid synthesis and oxidation are frequently exacerbated in leukemia cells, and may therefore represent a target for therapeutic intervention. In this work we analyzed the apoptotic and chemo-sensitizing action of the fatty acid oxidation inhibitor etomoxir in human acute myeloid leukemia cells. Etomoxir caused negligible lethality at concentrations up to 100 µM, but efficaciously cooperated to cause apoptosis with the anti-leukemic agent arsenic trioxide (ATO, Trisenox), and with lower efficacy with other anti-tumour drugs (etoposide, cisplatin), in HL60 cells. Etomoxir-ATO cooperation was also observed in NB4 human acute promyelocytic cells, but not in normal (non-tumour) mitogen-stimulated human peripheral blood lymphocytes. Biochemical determinations in HL60 cells indicated that etomoxir (25–200 µM) dose-dependently inhibited mitochondrial respiration while slightly stimulating glycolysis, and only caused marginal alterations in total ATP content and adenine nucleotide pool distribution. In addition, etomoxir caused oxidative stress (increase in intracellular reactive oxygen species accumulation, decrease in reduced glutathione content), as well as pro-apoptotic LKB-1/AMPK pathway activation, all of which may in part explain the chemo-sensitizing capacity of the drug. Etomoxir also cooperated with glycolytic inhibitors (2-deoxy-D-glucose, lonidamine) to induce apoptosis in HL60 cells, but not in NB4 cells. The combined etomoxir plus 2-deoxy-D-glucose treatment did not increase oxidative stress, caused moderate decrease in net ATP content, increased the AMP/ATP ratio with concomitant drop in energy charge, and caused defensive Akt and ERK kinase activation. Apoptosis generation by etomoxir plus 2-deoxy-D-glucose was further increased by co-incubation with ATO, which is apparently explained by the capacity of ATO to attenuate Akt and ERK activation. In summary, co-treatment with etomoxir may represent an interesting strategy to increase the apoptotic efficacy of ATO and (with some limitations) 2-deoxy-D-glucose which, although clinically important anti-tumour agents, exhibit low efficacy in monotherapy.

## Introduction

The acquisition of the tumor phenotype involves a profound re-organization of cellular metabolism, in such a manner that the altered metabolic pathways may represent potential targets for therapeutic intervention [1]–[3]. The best known example is the switch from oxidative phosphorylation to glycolysis as the main source of energy, even under aerobic conditions (aerobic glycolysis, or “Warburg effect”). No matter of the mechanisms responsible for this transformation, this property prompted the assay of anti-glycolytic agents, such as 2-deoxy-D-glucose (2-DG), lonidamine and 3-bromopyruvate (3-BrP), as potential anticancer agents, and promissory results have been obtained in clinical trials [4]–[6]. In addition to glycolytic addiction, tumour cells exhibit increased rates of fatty acid synthesis and glutamine utilization, which may also represent important therapeutic targets. In fact, fatty acid and glutamine metabolism provide necessary constituents to sustain rapid cell growth, and what is also important they may provide cancer cells with an alternative source of energy [2], [3], [7].

Etomoxir is an oxirane carboxylic acid derivative which irreversible inhibits carnitine palmitoyl transferase 1 (CPT1) activity [8], thus impeding fatty acid transport into mitochondria and further catabolism by β-oxidation. As a compensatory mechanism, inhibition of fatty acid oxidation may promote the energetically more efficient glucose oxidation. Because of this, etomoxir has been clinically used as a hypoglycemic drug against diabetes mellitus, and also to improve energy efficiency in chronic heart failure [9], [10]. In addition, later reports indicated that etomoxir triggers apoptosis, and what is probably more important, at low (sub-lethal) concentrations sensitizes to apoptosis induction by some conventional anti-tumour drugs [11]–[13], suggesting that it may represent a valuable tool in anticancer therapies. Nonetheless, this response is not universal [14], and what is more the mechanisms accounting for the pro-apoptotic action of etomoxir are not well understood. It has been proposed that fatty acid oxidation inhibition causes direct perturbations in Bcl-2 family proteins, which may explain potentiation of apoptosis by the Bcl-2 antagonist ABT-737 and perhaps other mitochondria-targeting drugs [12]. Other studies indicated that etomoxir causes ATP depletion as well as oxidative stress (increased reactive oxygen species (ROS) production and/or reduced glutathione (GSH) depletion), which are important determinants of cell death. However these responses were generally documented at high (lethal) drug concentrations [15]–[17], but not at low (sensitizing-inducing) concentrations [12]. Finally, the capacity of etomoxir to affect protein kinase pathways, important for apoptosis signaling, is almost unexplored.

Arsenic trioxide (ATO, Trisenox) is a clinically established drug for the treatment of acute promyelocytic leukemia (APL) [18]. At low concentrations (0.25–1.0 µM in plasma) the drug blocks cell growth and promotes terminal differentiation by disrupting the promyelocytic leukemia-retinoic acid receptor alpha (PML-RARα) oncogenic fusion protein, characteristic of APL. In addition, at higher concentrations ATO causes apoptosis in this and other cell types, a property which offers the possibility of extending the therapeutic application of the drug, especially against haematological malignancies [19], [20]. Nonetheless this latter approach would require the use of sensitizing strategies, to increase drug efficacy and reduce dosage to clinically achievable concentrations. As a part of a research program on the effects of metabolic inhibitors, we recently demonstrated the efficacy of the glycolytic inhibitors 2-DG, lonidamine and 3-BrP to cause apoptosis and/or augment the apoptotic efficacy of ATO and other anti-tumour drugs in leukemia cell models, but the results were discrepant in terms of drug potency, sensitization pattern, and death regulatory mechanisms [21]–[23]. In the present work we investigate the sensitizing capacity of etomoxir in HL60 cells, a human acute myeloid leukemia (AML) cell line characterized as poorly sensitive to ATO [24], and analyze the potential importance of alterations in energy pathways, oxidative stress and specific protein kinase activities as regulatory mechanisms. The obtained results demonstrate that etomoxir greatly potentiates ATO lethality, a response further improved by combination with glycolytic inhibitors. Generation of moderate oxidative stress and activation of the liver kinase B1/AMP-activated kinase (LKB-1/AMPK) pathway may in part explain the sensitizing capacity of etomoxir.

## Materials and Methods

### Reagents and antibodies

All components for cell culture were obtained from Invitrogen, Inc. (Carlsbad, CA). 4,6-diamino-2-phenylindole (DAPI) was obtained from Serva (Heidelberg, Germany), and dichlorodihydrofluorescein diacetate (H_2_DCFDA) and monochlorobimane from Molecular Probes, Inc. (Eugene, OR). The kinase inhibitors 6-[4(2-Piperidin-1-yl-ethoxy)phenyl)]-3-pyridin-4-yl-pyyrazolo[1,5-a] pyrimidine (Compound C), 1,4-Diamino-2,3-dicyano-1,4-*bis*(2-aminophenylthio)butadiene (U0126), 2′-amino-3′-methoxyflavone (PD 98059), 2-(4-Morpholinyl)-8-phenyl-4H-1-benzopyran-4-one (LY 294002), 5-Dihydro-5-methyl-1-β-D-ribofuranosyl-1,4,5,6,8-pentaazaacenaphtylen-3-amine hydrate (triciribine hydrate, Akt inhibitor V, Akt_i_V), and the caspase inhibitor Z-Val-Ala-Asp(OMe)-CH_2_F (z-VAD-fmk), were obtained from Calbiochem (Darmstad, Germany). Rabbit anti-human AMPKα, p44/42 MAP kinase, phospho-p44/p42 MAPK (Thr202/Tyr204), Akt, phospho-Akt (Ser473) (D9E) XP, and caspase-3 polyclonal antibodies; and rabbit anti-human phospho-AMPKα (Thr172) (40H9) and phospho-LKB1 (Ser428) (C6743) monoclonal antibodies, were obtained from Cell Signaling Technology Inc (Danvers, MA). Peroxidase-conjugated immunoglobulin G antibodies were from DAKO Diagnostics, S.A. (Barcelona, Spain). All other non-mentioned reagents and antibodies were from Sigma (Madrid, Spain).

### Cells and treatments

HL60 human myeloblastic leukemia cells [25] were obtained from our institutional repository (CIB), and PML-RARα-expressing NB4 human promyelocytic leukemia cells [26] were kindly provided by Profs. J. León and M.D. Delgado (Departamento de Biología Molecular, Facultad de Medicina, Universidad de Cantabria, Santander, Spain). Authentication by STR analysis, specific antigen expression and other functional markers, as well as the absence of mycoplasma contamination, was corroborated by us or our technical staff. Human peripheral blood lymphocytes (PBLs) from healthy voluntary donors were obtained from buffy-coats supplied by the blood bank of the “Centro de Transfusion de la Comunidad de Madrid”. The samples were provided without any information about the donor, ant tested to ensure the absence of pathogens. The whole procedure, including the use of leukemia cell lines and PBLs, was approved by the Bioethics and Biosafety Commission of our Institution (Centro de Investigaciones Biológicas, CSIC). Conditions of cell growth and treatment, including PBLs preparation and mitogenic stimulation with phytohemagglutinin/interleukin-2, were already described in detail in preceding publications [21], [22]. Stock solutions of orlistat (40 mM), lonidamine (100 mM), etoposide (20 mM) H_2_DCFDA (5 mM), Compound C, U0126, PD 98059, LY 294002 and Akt_i_V (20 mM each), z-VAD-fmk (25 mM), oligomycin (1 mM) and monochlorobimane (200 mM), were prepared in dimethyl sulfoxide. 3(4,5-Dimethyl-2-thiazolyl)-2,5-diphenyl-2*H*-tetrazolium bromide (MTT) was dissolved at 5 mg/ml in PBS, and oligomycin in RPMI 1640 at 31.6 mM. Stock solutions of etomoxir (10 mM) and cis-platinum(II)-diammine dichloride (cisplatin, CDDP, 3.3 mM) were prepared in distilled water. All these solutions were stored at −20°C. Stock solutions of DAPI (10 µg/ml) and propidium iodide (PI, 1 mg/ml) were prepared in phosphate buffered saline (PBS). ATO was initially dissolved in a small amount of 1 N NaOH, and then diluted with PBS to give a final concentration of 10 mM. These solutions were stored at 4°C. 2-DG was freshly prepared at 250 mM in PBS. 3-BrP was freshly prepared at 30 mM in PBS, and the pH of the solution was adjusted at 7.2 with NaOH.

### Flow cytometry

The analysis of samples was carried out on an EPICS XL flow cytometer (Coulter, Hialeah, FL) equipped with an air-cooled argon laser tuned to 488 nm. The specific fluorescence signal corresponding to fluorescein isothiocyanate and H_2_DCFDA was collected with a 525-nm band pass filter, and the signal corresponding to PI with a 620-nm band pass filter. A total of 10^4^ cells were scored in cell cycle assays, and 5×10^3^ cells in the H_2_DCFDA assays.

### Cell viability, cell cycle, apoptosis and necrosis

Cell viability was normally determined by the MTT colorimetric assay. This procedure gives an indirect estimation of the relative number of viable cells in the culture, based on changes in mitochondrial metabolic activity. However in the case of etoposide and cisplatin, which cause cell hypertrophy with increased amount of mitochondria per cell, proliferation was measured by direct cell counting. Cell cycle phase distribution was routinely determined by cell permeabilization followed by PI staining and flow cytometry analysis, and the histograms analyzed with the Cyflogic analysis program (CyFlo Ltd., Turku, Finland). This technique also provided an estimation of the frequency of apoptotic cells, characterized by low (sub-G_1_) DNA content. In all experiments, apoptosis was also evaluated as the fraction of cells with condensed/fragmented chromatin, and occasionally chromatin loss, as determined by cell permeabilization followed by DAPI staining and microscopy examination. The criterion used for necrosis (either genuine, “primary” necrosis or apoptosis-derived, “secondary” necrosis) was the loss of plasma membrane integrity, as determined by free PI uptake into non-permeabilized cells and flow cytometry analysis. A detailed description of all these techniques can be found in our preceding works ([27], [28] and references therein), and hence is omitted here.

### Oxygen consumption and rate of extracellular acidification

The oxygen consumption rate (OCR) and the extracellular acidification rate (ECAR, a proxy for lactate production) were simultaneously determined using an XF24 Seahorse Bioscience instrument (North Billerica, MA) (Wu et al., 2007). The adaptation of the procedure to HL60 cells was described in detail in our preceding work [23].

### ATP, ADP and AMP levels

The total intracellular ATP content was quantified using the ATP Bioluminescence Assay Kit ASII (Roche, Mannheim, Germany), which measures ATP based on a luciferase-catalized luciferin oxidation reaction. Samples of approximately 10^6^ cells were washed once with PBS and then processed according to the manufacturer's protocol. The ATP-derived fluorescent signal was measured on a Varioskan Flash microplate reader (Thermo Fisher Scientific Inc, Waltham, MA). Cell samples were collected in parallel to take into account possible variations in protein content, and the ATP values were normalized accordingly.

The relative adenine nucleotide (AMP, ADP and ATP) pools were determined by reverse-phase high performance liquid chromatography (HPLC) essentially as described by De Korte et al. [29]. 4×10^6^ cells were spun for 30 seconds at 16,000×*g*, the supernatant removed and the pellet rapidly quenched and re-suspended in 660 mM HClO_4_ and 10 mM theophylline. Extracts were centrifuged 15 min at 16,000×*g* and the supernatants neutralized using 2.8 M K_3_PO_4_. Analytical separation was performed using a Shimadzu Prominence chromatograph (Canby, OR) equipped with a C18 column (Mediterranea SEA 18, Teknokroma, Sant Cugat, Spain). Peak identities were confirmed by comparison with the retention times of standard adenine nucleotides, and extracts from cells exposed to 1 µM oligomycin plus 30 mM 2-DG were used to validate the technique (S1 Figure).

### ROS and GSH levels

The intracellular accumulation of ROS was measured by flow cytometry using the ROS-sensitive probe H_2_DCFDA. The intracellular GSH content was determined in a Varioskan Flash microplate reader at excitation wavelength of 390 nm and emission wavelength of 520 nm, using the fluorescent probe monochlorobimane. The detailed protocols were already described in a previous publication [23].

### Immunoblotting

Cells were collected by centrifugation, washed with PBS and total protein extracts were obtained by lysing them for 20 min at 4°C in a buffer consisting of 20 mM Tris-HCl (pH 7.5) containing 137 mM NaCl, 2 mM EDTA, 10% (v/v) glycerol, and 1% Nonidet P-40, and supplemented with a protease inhibitor cocktail, 1 mM sodium orthovanadate, and 10 mM NaF. After brief sonication and centrifugation for 15 min at 10,000×*g* at 4°C, the supernatants were collected, and samples containing equal amounts of proteins were resolved by SDS-polyacrylamide gel electrophoresis. The proteins were then transferred to polyvinylidene fluoride (PVDF) membranes and immunodetected, as previously described [30]. When convenient, the relative band intensities were quantified using the Quantity One 1-D Analysis Software, version 4.6 (Bio-Rad Laboratories, Inc, Hercules, CA).

### Data analysis and presentation

Except when indicated, all experiments were repeated at least three times, and as a rule the results are expressed as the mean value ± SD. Statistical analyses were carried out using one way ANOVA with Dunnet or Bonferroni post-test, using the GraphPad Prism (version 5.00) software (GraphPad software, Inc, LaJolla CA). The symbols used were: *, to compare treatment vs. control, or pairs of treatments; and ^#^, to indicate that the value in a combined treatment is higher than the sum of values in the corresponding single treatments. In all cases, single symbol means p<0.05, double symbol p<0.01, and triple symbol p<0.001. n.s., non-significant. In some cases, drug interaction was also examined by calculating the combination index (CI), using the Compusyn software (Combosyn, Inc, Paramus, NJ), based on the Chou and Talalay procedure [31]. CI values below 1.0 indicate synergism, values around 1.0 additive effect, and values above 1.0 antagonism.

## Results

### Cell viability, cell cycle and apoptosis

**Figure 1:**
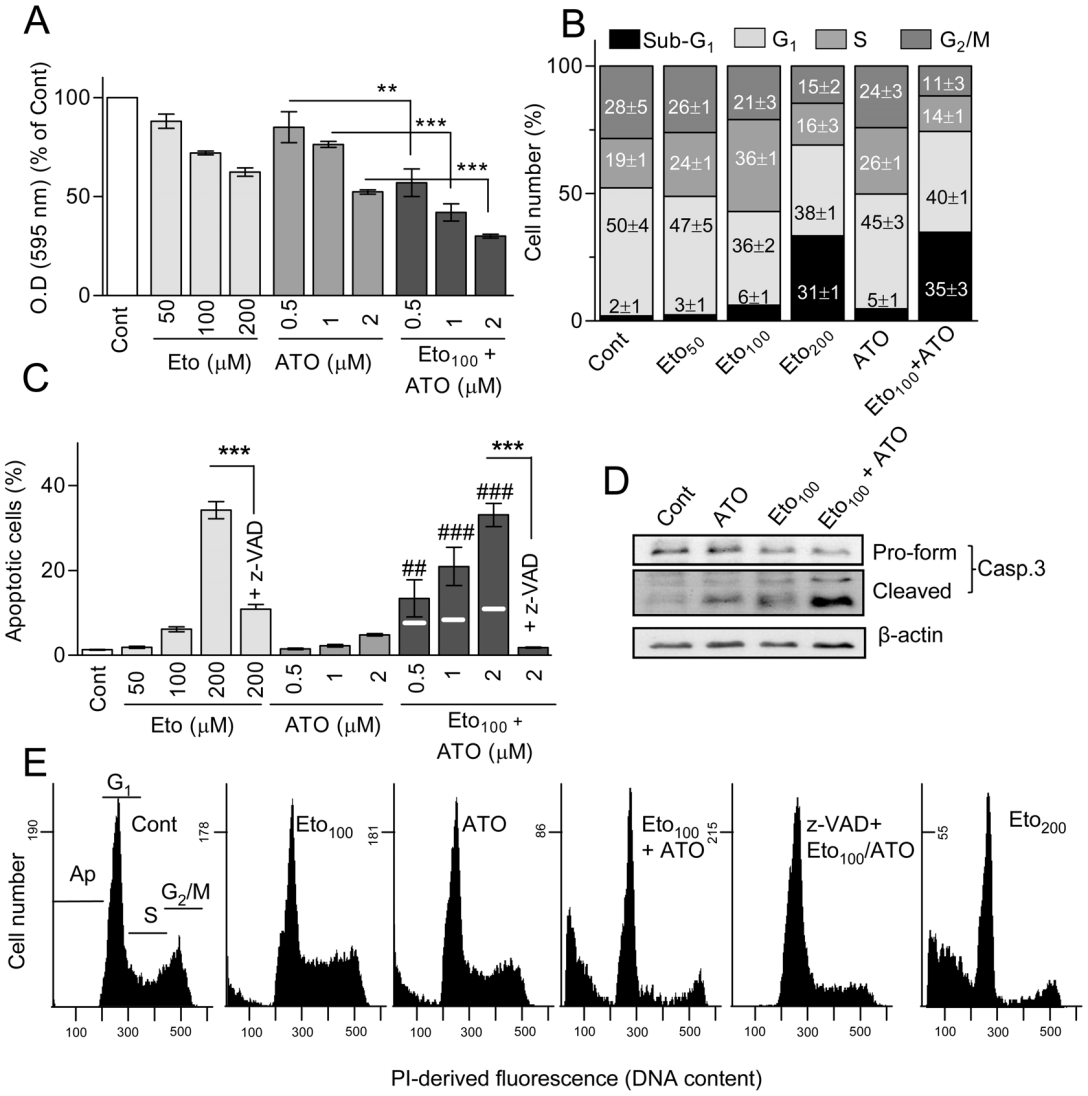
Effect of etomoxir and ATO on cell viability, cycle phase distribution and apoptosis generation in HL60 cells. Cells were incubated for 24 h with the indicated concentrations of etomoxir (Eto) and ATO, alone and in combination. When nothing is indicated, ATO was used at 2 µM. For convenience, the drug concentrations are sometimes indicated as subheadings. When indicated, the cells were co-incubated with the pan-caspase inhibitor z-VAD-fmk (50 µM). (A) Changes in cell viability, as evidenced by the MTT assay. Absorption values are indicated in relation to untreated (Cont) cultures. (B) Frequency of cells at the different phases of the growth cycle, namely G_1_, S and G_2_/M, and with sub-G_1_ DNA content (apoptotic). Examples of flow cytometry histograms are presented in (E). (C) Frequency of apoptotic cells, as determined by flow cytometry. (D) Caspase-3 cleavage/activation, determined by immunoblot. β-actin is included as a loading control. The bar charts in (A–C) represent the mean ± S.D. of at least three determinations. Symbols mean: (*) significant differences between the indicated pair values; (^#^) significant differences between the combined treatment and the sum of values in the corresponding individual treatments (e.g., co-incubation with 100 µM Eto and 2 µM ATO, in relation to the sum of 100 µM Eto alone plus 2 µM ATO alone) (n.s., non-significant). To better discern differences, in this case the sum of values in individual treatments is indicated by a horizontal white line within the bar corresponding to the combined treatment. Single symbol, *p*<0.05; double symbol, *p*<0.01; triple symbol, *p*<0.001.

We firstly examined the capacity of etomoxir, alone and in combination with the anti-leukemic agent ATO, to affect viability and cycle phase distribution, and to cause apoptosis in HL60 cells. Etomoxir was assayed at concentrations of 50–200 µM, on the ground of earlier studies in leukemia and myeloma cells [12], [14]. ATO was assayed at 0.5–2 µM, which are considered clinically relevant concentrations [32]. Cell viability was determined by the MTT assay, and cell cycle alterations by PI staining and flow cytometry. Apoptosis was estimated as the frequency of cells with sub-G_1_ DNA content in the flow cytometry assays, and the results were always qualitatively corroborated by examining chromatin condensation/fragmentation. The results, some of which are represented in Fig. 1, may be summarized as follows: (i) Incubation with 50–200 µM etomoxir caused dose-dependent decrease in the number of viable cells (Fig. 1A). At 50–100 µM the drug caused cell cycle perturbation, consisting in accumulation of cells at S-phase (Fig. 1B, E), but apoptosis induction was very low (less than 10%) (Fig. 1B, C). The drug moderately induced apoptosis at 200 µM (30–35% apoptotic cells) (Fig. 1C, E). (ii) Incubation for 24 h with 0.5–2 µM ATO caused concentration-dependent decrease in cell viability (Fig. 1A), but little (less than 10%) apoptotic effect (Fig. 1B, C). (iii) Co-incubation with etomoxir augmented ATO-provoked viability decrease (Fig. 1A), and also potentiated apoptosis generation reaching approximately 30–35% apoptotic cells with 100 µM etomoxir and 2 µM ATO (Figs. 1C, E and S2B Figure). Drug cooperation was initially estimated as more than additive (Fig. 1C), and synergism could be demonstrated by CI calculation (CI values of 0.54 and 0.35, using 100 µM etomoxir plus 1 and 2 µM ATO, respectively). Time-course determinations with 100 µM etomoxir plus 2 µM ATO indicated 5.1±1.1%, 8.2±1.9%, and 15.3±3.7% apoptotic cells at 4, 8 and 16 h, respectively, and on this ground 8 h was the maximum treatment time selected for further studies of regulatory mechanisms. (iv) Etomoxir plus ATO caused caspase-3 cleavage/activation (Fig. 1D), and the lethality of 200 µM etomoxir alone or 100 µM etomoxir plus 2 µM ATO was decreased by the pan-caspase inhibitor z-VAD-fmk (Fig. 1C), indicating bona fide caspase-dependent apoptosis. (v) In addition to the expression of apoptotic markers, etomoxir plus ATO caused plasma membrane damage in a small fraction of cells, as evidenced by free PI penetration, but this fraction was almost suppressed by co-treatment with z-VAD-fmk (11.9±3.2% vs. 2.3±1.2% in the absence and presence of the caspase inhibitor, respectively, at 24 h of treatment with 100 µM etomoxir plus 2 µM ATO). Hence, plasma membrane permeability likely represents late apoptosis instead of genuine necrotic response.

**Figure 2:**
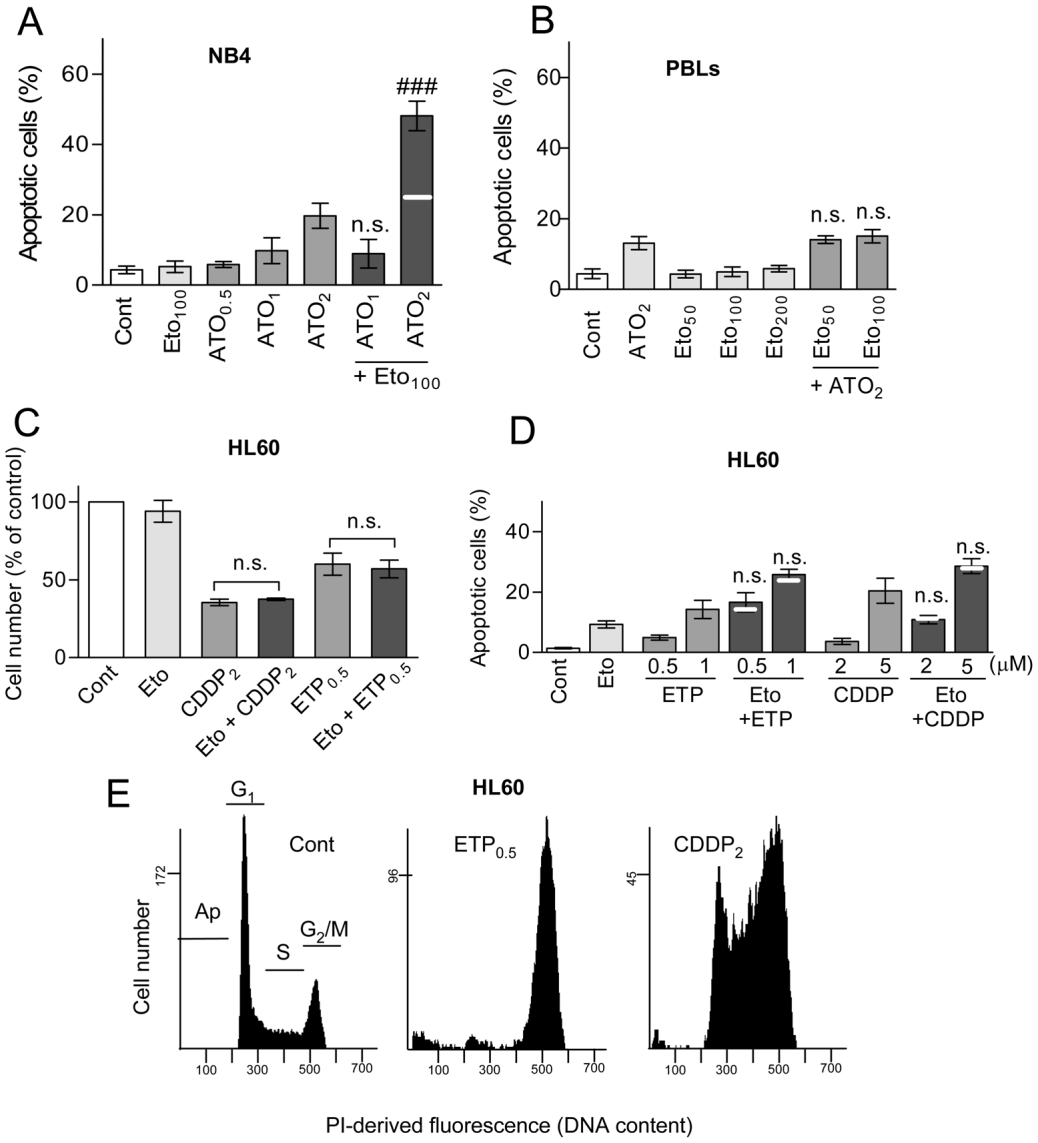
Effect of etomoxir, etoposide and cisplatin on cell proliferation, cycle phase distribution and apoptosis generation in different cell models. Cells were incubated for 24 h with the indicated concentrations of etomoxir (Eto), ATO, etoposide (ETP) or cisplatin (CDDP), alone or in combination. When nothing is indicated, etomoxir was used at 100 µM. (A) Apoptosis generation, measured by flow cytometry, in NB4 cells. (B) The same, in mitogen-stimulated human PBLs. (C, D) Changes in proliferation activity, measured by cell counting (C), and apoptosis generation, measured by flow cytometry (D), in HL60 cells. Changes in proliferation are expressed in relation to untreated (Cont) cultures. (E) Flow cytometry histograms showing changes in cycle distribution in HL60 cell cultures treated with etoposide and cisplatin. The bar charts in (A–D) represent the mean ± S.D. of at least three determinations. Symbols and n.s. indicate significant and non-significant differences, respectively, between the indicated pairs of values (C), or between the combined treatment and the sum of values in the corresponding individual treatments (A, B, D). For other conditions see legend of Fig. 1.

In addition, we examined the potential cooperation between etomoxir and ATO in other cell models, namely NB4 APL cells and mitogen-stimulated non-tumour PBLs, as well as the potential cooperation between etomoxir and antitumour drugs other than ATO, namely the DNA topoisomerase II poison etoposide and the DNA alkylating agent cisplatin, in HL60 cells. The results were as follows: (i) NB4 cells were similarly sensitive to apoptosis induction by etomoxir as HL60 cells, but more sensitive to apoptosis induction by ATO (approximate apoptotic rate of 20% at 24 h incubation with 2 µM), as earlier described [24]. Etomoxir and ATO cooperated in more than additive manner to cause apoptosis in NB4 cells, but only at the higher ATO concentration (Fig. 2A). PBLs were resistant to etomoxir and slightly sensitive to ATO, and the combination produced null cooperative effect (Fig. 2B) (ii) Etoposide and cisplatin decreased cell proliferation in HL60 cells (Fig. 2C), concomitantly with cycle arrest at G_2_/M (etoposide) or S plus G_2_/M (cisplatin) (Fig. 2E), and caused cell hypertrophy (not shown). Co-incubation with etomoxir did not augment proliferation inhibition (Fig. 2C), although it increased slightly apoptosis induction by etoposide (0.5 and 1 µM) and cisplatin (2 and 5 µM) (Fig. 2D). Nonetheless, this increase was of lower magnitude (approximately additive) than in the case of etomoxir plus ATO. Of note, the lower apoptotic efficacy was not explained by a switch to a necrotic response, since the frequency of PI permeable cells always remained below 10% within the 24 h treatment period (data not shown).

### Energy metabolism and oxidative stress

**Figure 3:**
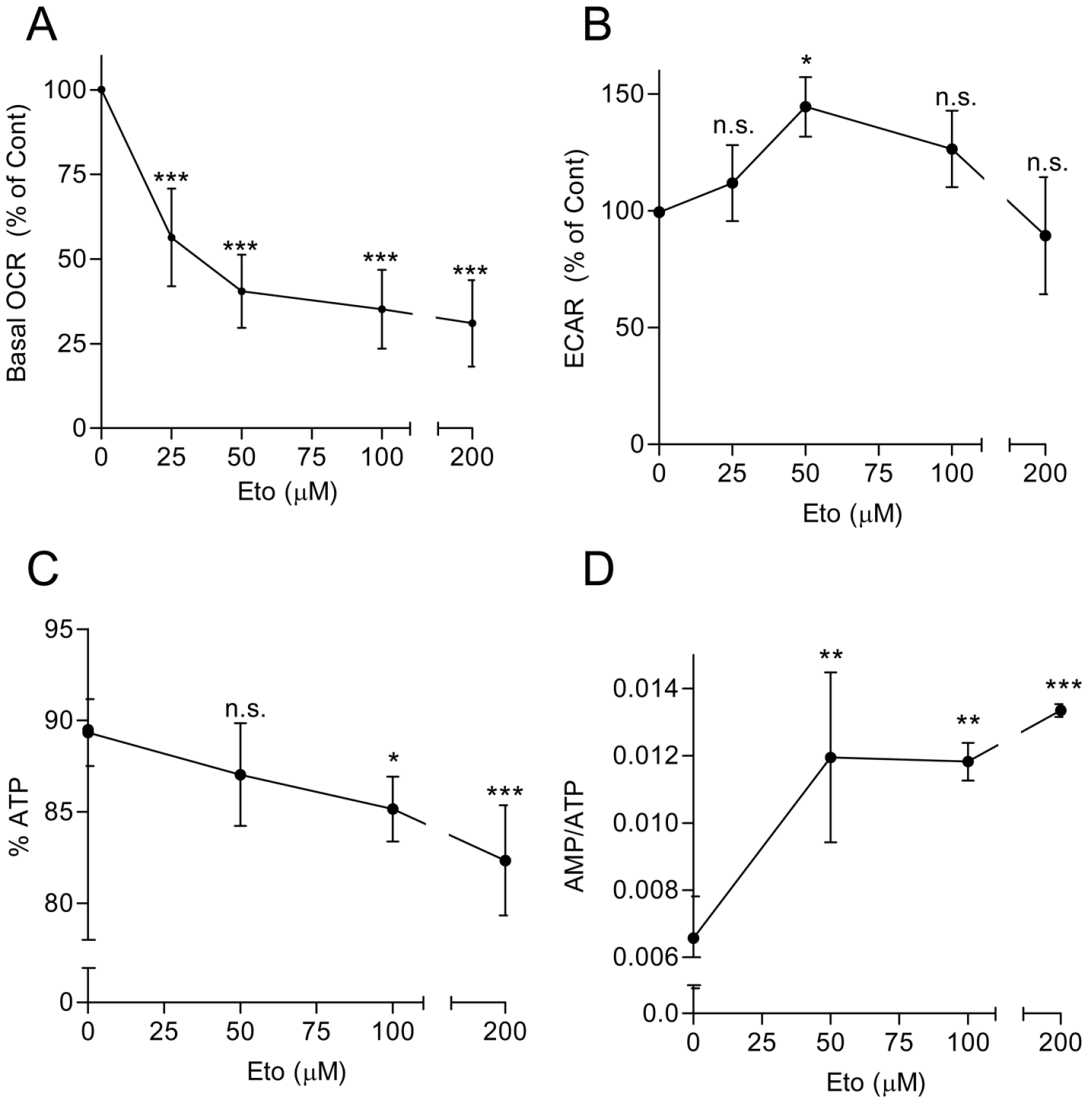
Effect of etomoxir on energy metabolism in HL60 cells. (A, B) The basal rates of oxygen consumption (OCR) and extracellular acidification (ECAR, a proxy for lactate formation) were simultaneously determined upon 1 h exposure to the indicated concentrations of etomoxir on a Seahorse XF24 metabolic flux analyzer. Data points represent the mean ± S.D. of six independent experiments performed in sextuplicate. The results are expressed in relation to the control (OCR: 287.1±26.3 pmol O_2_/min; ECAR: 26.8±3.2 mpH units/min). (C, D) The relative fraction of ATP in relation to total nucleotide (ATP+ADP+AMP) content (C), and the AMP/ATP ratio (D), were determined by HPLC upon 2 h exposure to the indicated concentrations of etomoxir. Data point represents the mean ± S.D. of at least four independent experiments. For more complete information see S1 Table. Symbols indicate significant differences in relation to drug-untreated cells (0 µM) (n.s., non-significant). For other conditions see legend of Fig. 1.

It was reported that etomoxir readily inhibits mitochondrial respiration [12], [17], and at elevated concentrations causes ATP depletion [15]–[17]. ATP depletion may be a determinant for cell death, either apoptosis or necrosis, depending on the magnitude of the decrease [33], [34]. For these reasons, experiments were carried out to simultaneously measure OCR and ECAR (a proxy for lactate formation) as an estimate of the rates of mitochondrial respiration and glycolysis, respectively, as well as the net ATP content and the relative distribution of adenine nucleotides in HL60 cells. It was found that 25–200 µM etomoxir dose-dependently inhibited mitochondrial respiration activity (Fig. 3A), with a concomitant slight increase in lactate formation (at the concentrations of 50–100 µM) (Fig. 3B). Determinations of net ATP content by bioluminiscence assay indicated null effects of 25–100 µM etomoxir, and an approximate 20% decrease at 200 µM, at 4 h of treatment (S3A Figure). Determinations of adenine nucleotide levels by HPLC showed that 2–6 h exposure to 50–200 µM etomoxir caused slight, concentration-dependent reduction in the relative ATP fraction paralleled by slight increases in the relative ADP and AMP fractions, and consequently in AMP/ATP ratio (Fig. 3C, D, and S1 Table).

**Figure 4:**
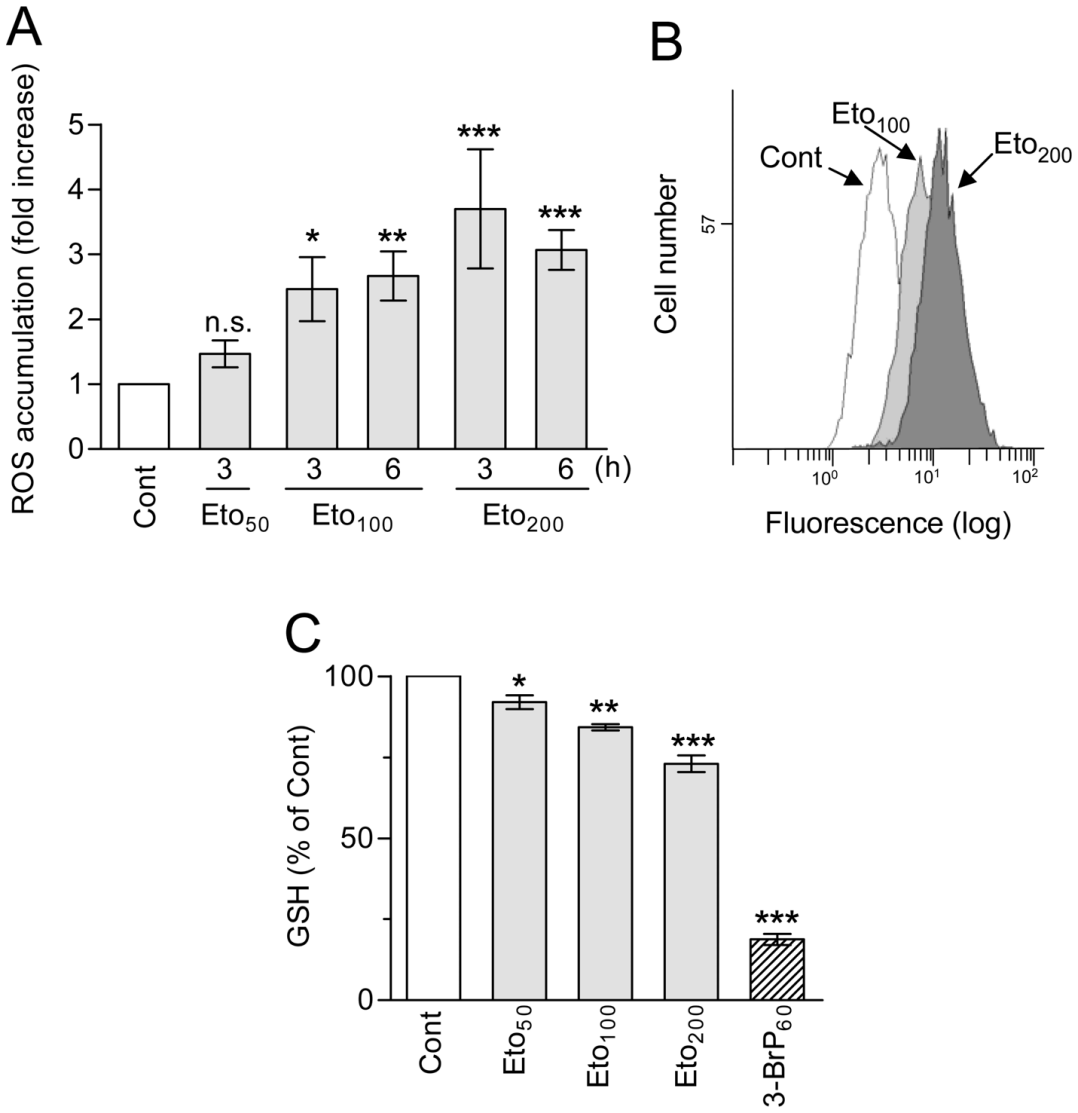
Effect of etomoxir on ROS and GSH levels in HL60 cells. (A, B) Intracellular accumulation of ROS, as determined by flow cytometry using H_2_DCFDA. The results are expressed as fold increase in relation to untreated (Cont) cells. Histograms corresponding to untreated cells and cells incubated for 3 h with 100 and 200 µM etomoxir are shown as examples. (C) Intracellular GSH levels, as determined at 4 h of incubation by monochlorobimane derivatization in luminometric assays. 3-BrP (60 µM) was used as a positive control (see Ref. 23). The results are expressed in relation to untreated (Cont) cells (approximate GSH content, 8.5 nmol/10^6^ cells). The values represent the mean ± S.D. of at least three determinations. Symbols indicate significant differences in relation to Cont (n.s., non significant). For other conditions see legend of Fig. 1.

It was reported that etomoxir may cause oxidative stress, as indicated by ROS overproduction and/or GSH depletion [15]–[17], [35]. In addition, the intracellular oxidant state is an important determinant of ATO toxicity [36], [37]. For these reasons, we analyzed possible alterations in intracellular ROS accumulation and GSH levels in etomoxir-treated HL60 cells. It was observed that incubation for 3–6 h with 50–200 µM etomoxir dose-dependently induced ROS over-accumulation, as measured by the increase in H_2_DCFDA-derived fluorescence (Fig. 4A, B). In addition, upon 4 h incubation the drug caused a slight, dose-dependent decrease in intracellular GSH content (Fig. 4C).

### Protein kinase activities

**Figure 5:**
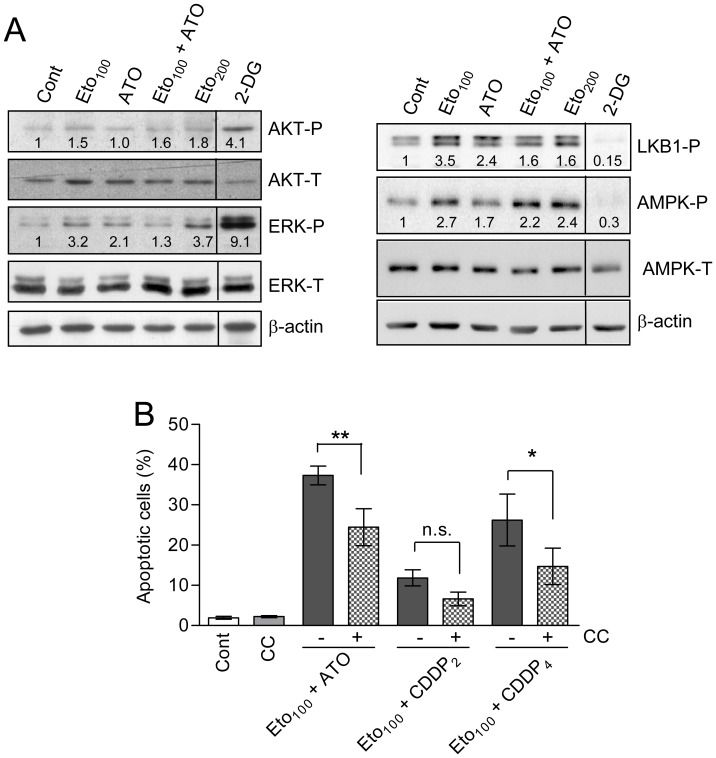
Effect of etomoxir on protein kinase activities, and effect of AMPK inhibitor, in HL60 cells. (A) Relative levels of phosphorylated (P) and total (T) Akt, ERKs, LKB1 and AMPK upon incubation for 4 h with etomoxir and ATO, alone and in combination. Extracts from cells incubated with 10 mM 2-DG, used as a positive control (see Ref. 21), were run in a parallel lane within the same gel. The level of β-actin was assessed as a control of sample loading. The numbers inside the blots indicate the values obtained by quantification of band intensities, once normalized in relation to the corresponding β-actin level, and expressed in relation to the Control (which received the arbitrary value of one). The blots are representative of one out of four independent experiments, with qualitatively similar results. (B) Apoptosis generation at 24 h of incubation with etomoxir plus either ATO or cisplatin, alone (−) or in combination (+) with the AMPK inhibitor compound C (CC, 7 µM). The values are the mean ± S.D. of four determinations. Symbols indicate significant differences between the indicated pairs of values (n.s., non-significant). ATO was always used at 2 µM. For other conditions see legend of Fig. 1.

We recently reported that the glycolytic inhibitors 2-DG and lonidamine activated protein kinase B (Akt) and extracellular-signal regulated kinases (ERK) in AML cells, representing a defensive response which reduces drug lethality, but they caused drug-dependent differential effects (either inhibition or activation) on the of LKB-1/AMPK pathway [21], [22]. Since the action of fatty acid inhibitors on these signaling pathways is little known, we examined the capacity of etomoxir (100 and 200 µM), alone and with ATO, to modulate the phosphorylation/activation state of these kinases. A representative blot obtained at 4 h of incubation is shown in Fig. 5A. It was observed that etomoxir caused marginal effect on Akt phosphorylation, but produced an appreciable increase in ERK phosphorylation as well as in LKB-1 and AMPK phosphorylation. Similar results were obtained at 8 h of incubation (data not shown). 2-DG (10 mM), included as a control, strongly stimulated Akt and ERK phosphorylation but reduced LKB-1 and AMPK basal phosphorylation, as expected [22].

ERK behaves as a defensive kinase [21], and hence its activation may not explain apoptosis potentiation by etomoxir. On the other hand, AMPK may either play pro-apoptotic or survival roles [38], [39]. For this reason, we examined the effect of the AMPK-specific inhibitor compound C (7 µM) on drug lethality. The capacity of the inhibitor to effectively prevent kinase activation in HL60 cells was corroborated in a previous work [22]. As indicated in Fig. 5B and S2E Figure, compound C attenuated the generation of apoptosis by etomoxir plus ATO and etomoxir plus cisplatin. This indicates that in the present experimental conditions AMPK activation plays a pro-apoptotic role, and hence may in part explain the increased lethality of the combinatory treatments.

### Combination of etomoxir with glycolysis inhibitors

**Figure 6:**
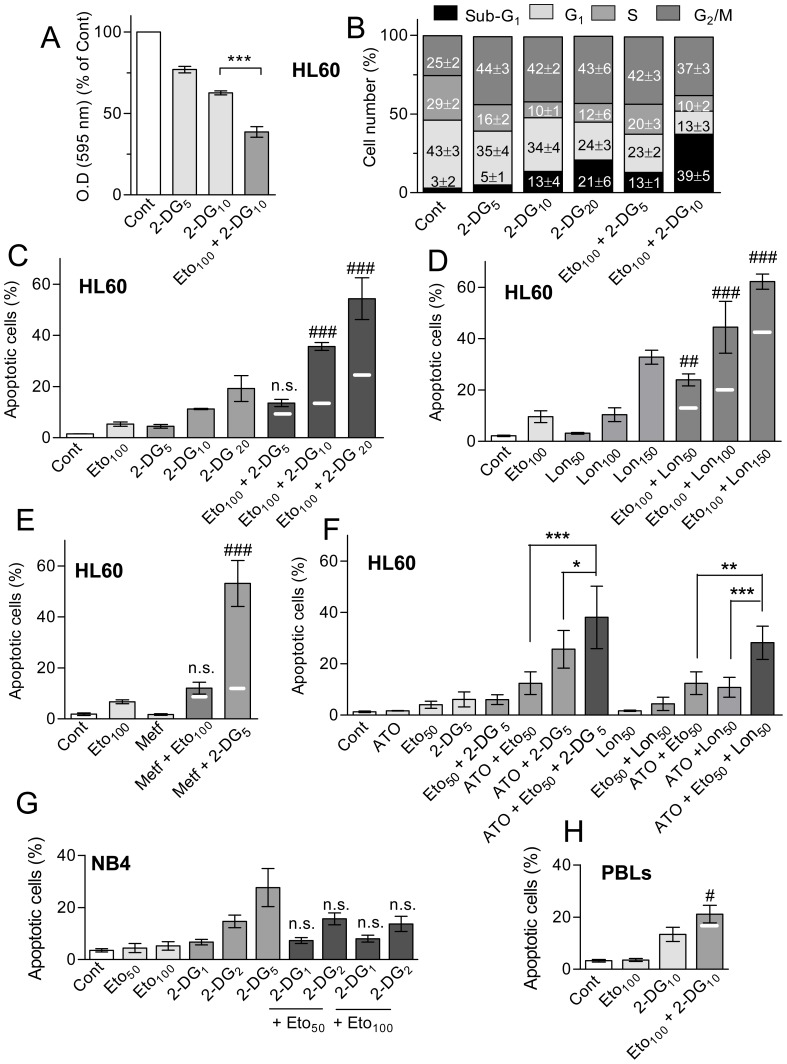
Effects of etomoxir in combination with other metabolic inhibitors, in different cell models. (A) Changes in viability, as evidenced by the MTT assay, in HL60 cells treated with 5 and 10 mM 2-DG, alone and in combination with 100 µM etomoxir. Absorption values are indicated in relation to untreated (Cont) cultures. (B) Frequency of HL60 cells at the different growth cycle phases and with sub-G_1_ DNA content (apoptotic) upon incubation with 5–20 mM 2-DG, alone or with 100 µM etomoxir. (C–E) Frequency of apoptosis in HL60 cell cultures upon incubation with: (C) 100 µM etomoxir and 5–20 mM 2-DG, alone or in combination; (D) the same, using etomoxir and lonidamine (Lon, 50–150 µM); (E) the same, using etomoxir and metformin (Metf, 4 mM). Metformin plus 2-DG was included as a positive control. (F) Frequency of apoptosis in HL60 cell cultures incubated with 2 µM ATO in combination with 50 µM etomoxir plus 5 mM 2-DG, in relation to ATO plus 2-DG alone and ATO plus etomoxir alone; and in cultures incubated with 2 µM ATO in combination with 50 µM etomoxir plus 50 µM lonidamine, in relation to ATO plus lonidamine alone and ATO plus etomoxir alone. (G) Frequency of apoptosis in NBA cell cultures treated with 1–5 mM 2-DG, alone and in combination with 50–100 µM etomoxir. (H) The same in mitogen-stimulated PBLs, using 10 mM 2-DG and 100 µM etomoxir. All treatments lasted for 24 h. All values are the mean ± S.D. of at least four determinations. Symbols in A and F indicate significant differences between the indicated pairs of values. Symbols in C, D, E, G, H, indicate significant differences between the combined treatment and the corresponding individual treatments (n.s., non-significant). For other conditions see legend of Fig. 1.

Glycolysis and mitochondrial oxidative phosphorylation represent alternative energy supply pathways which may mutually compensate [8], [40]. Since etomoxir inhibited mitochondrial respiration with occasional activation of lactate production (see Fig. 3), we asked whether a more efficacious apoptotic response could be obtained by simultaneously targeting the two pathways. Preliminary experiments indicated that incubation of HL60 cells with 5–10 mM 2-DG caused concentration-dependent decrease in viability, which was augmented by co-incubation etomoxir (Fig. 6A), and caused also cell cycle disruption characterized by cell accumulation at G_2_/M, with concomitant reduction in S phase and G_1_ (Fig. 6B). 2-DG alone caused little (lower than 10%) apoptosis at concentrations below 20 mM, but efficaciously cooperated with etomoxir to induce apoptosis in more than additive, synergistic manner (Fig. 6C and S2C Figure) (CI values of 0.45 and 0.37, using 100 µM etomoxir and 10 and 20 mM 2-DG, respectively). Apoptosis was potentiated with similar efficacy using the anti-glycolytic agent lonidamine instead of 2-DG (Fig. 6D) (CI values of 0.66 and 0.65 using 100 µM etomoxir and 100 and 150 µM lonidamine, respectively). For comparison, experiments were carried out using metformin, which inhibited mitochondrial respiration while enhancing glycolysis in HL60 cells (OCR value of 37% and ECAR value of 200% in relation to the control, upon 2 h treatment with 4 mM metformin). Es indicated in Fig. 6E, the benefit of combining etomoxir with metformin was very low, while treatment with metformin plus 2-DG, used as an internal control, was highly toxic, as expected [41]. Of note, we could not observe beneficial cooperation between 2-DG and etomoxir in NB4 cells, a cell line described as highly dependent on glycolysis [42] and consequently with higher sensitivity to 2-DG than HL60 cells (e.g., nearly 30% apoptosis upon treatment with 5 mM 2-DG: Fig. 6G). Concerning mitogen-stimulated PBLs, we could observe cooperation slightly higher than additive to induce apoptosis between 100 µM etomoxir and 10 mM 2-DG (Fig. 6H), although the intensity of cooperation was much lower than in the case of HL60 cells (see Fig. 6C). Finally, we wanted to know whether combining etomoxir with 2-DG could be also beneficial in terms of cooperation with ATO. In these experiments the concentration of etomoxir was reduced to 50 µM, to minimize the basal lethality of etomoxir plus 2-DG combination. It was observed that the rate of apoptosis obtained with the triple combination (i.e., ATO with etomoxir plus 2-DG) was clearly higher than that obtained by any double combination (i.e., ATO with etomoxir, or ATO with 2-DG) in HL60 cells (Fig. 6F and S2D Figure). Similar results were obtained in the triple combination assay, using lonidamine instead of 2-DG (Fig. 6F).

**Figure 7:**
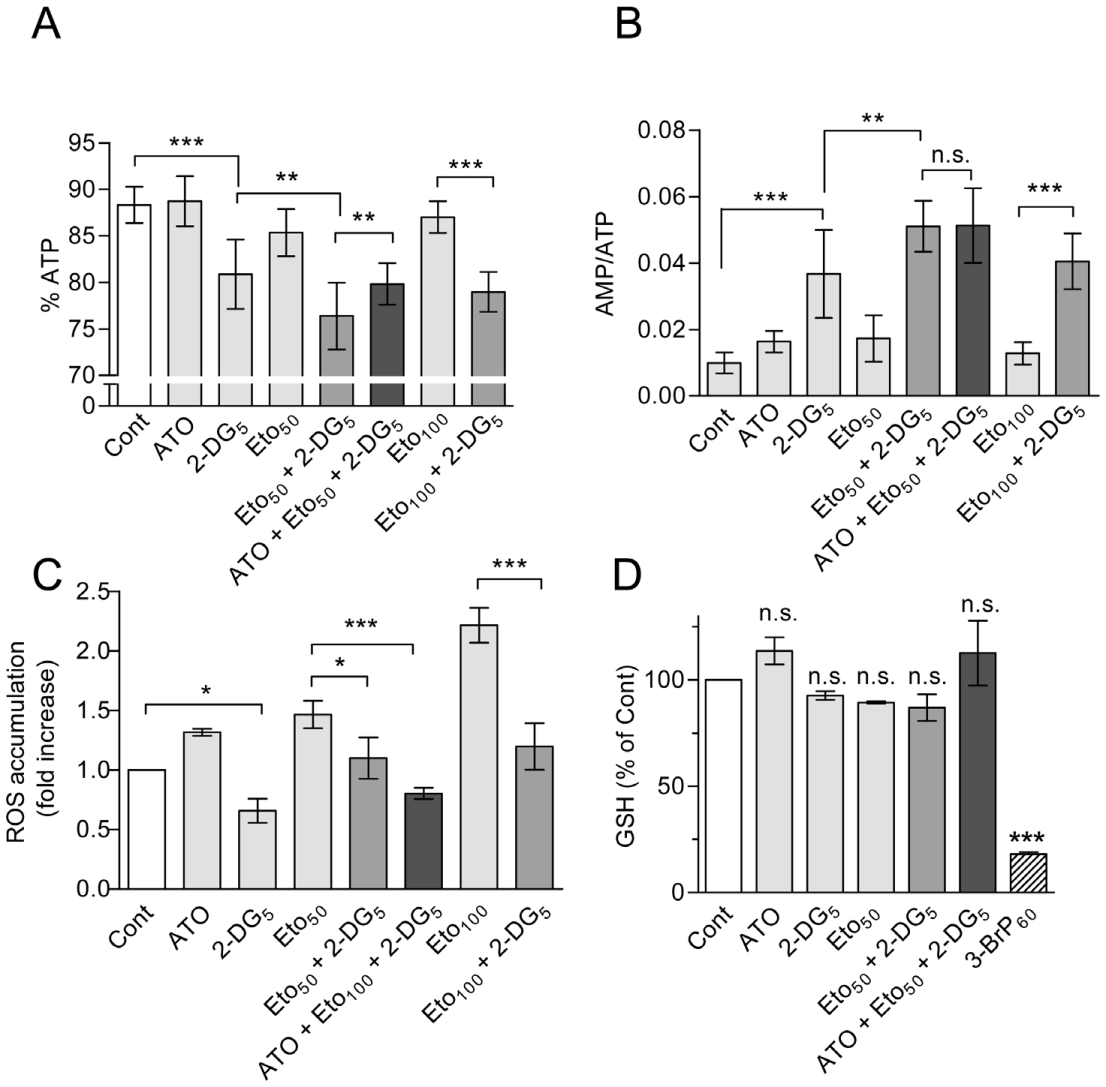
Energy metabolism and oxidative stress upon incubation with etomoxir, 2-DG and ATO in HL60 cells. (A, B) Relative fraction of ATP in relation to total nucleotide (ATP+ADP+AMP) content (A), and AMP/ATP ratio (B), in untreated cells (Cont) and cells incubated for 6 h with the indicated concentrations of 2-DG and etomoxir, alone and in combination, and in the presence or absence of ATO. For more complete information see S1 Table. (C, D) Changes in intracellular ROS accumulation (C) and GSH content (D), in cells incubated for 4 h with etomoxir and 2-DG, alone or in combination, and in the presence or absence of ATO. 3-BrP (60 µM) was used as a positive control in D (see Ref. 23). The values represent the mean ± S.D. of at least five determinations. Symbols in A–C indicate significant differences in relation to the control or between the indicated pairs of treatments. Symbols in D indicate significant differences in relation to the control (n.s., non-significant). ATO was always used at 2 µM. For other conditions, see legends of Figs. 1 and 4.

**Figure 8:**
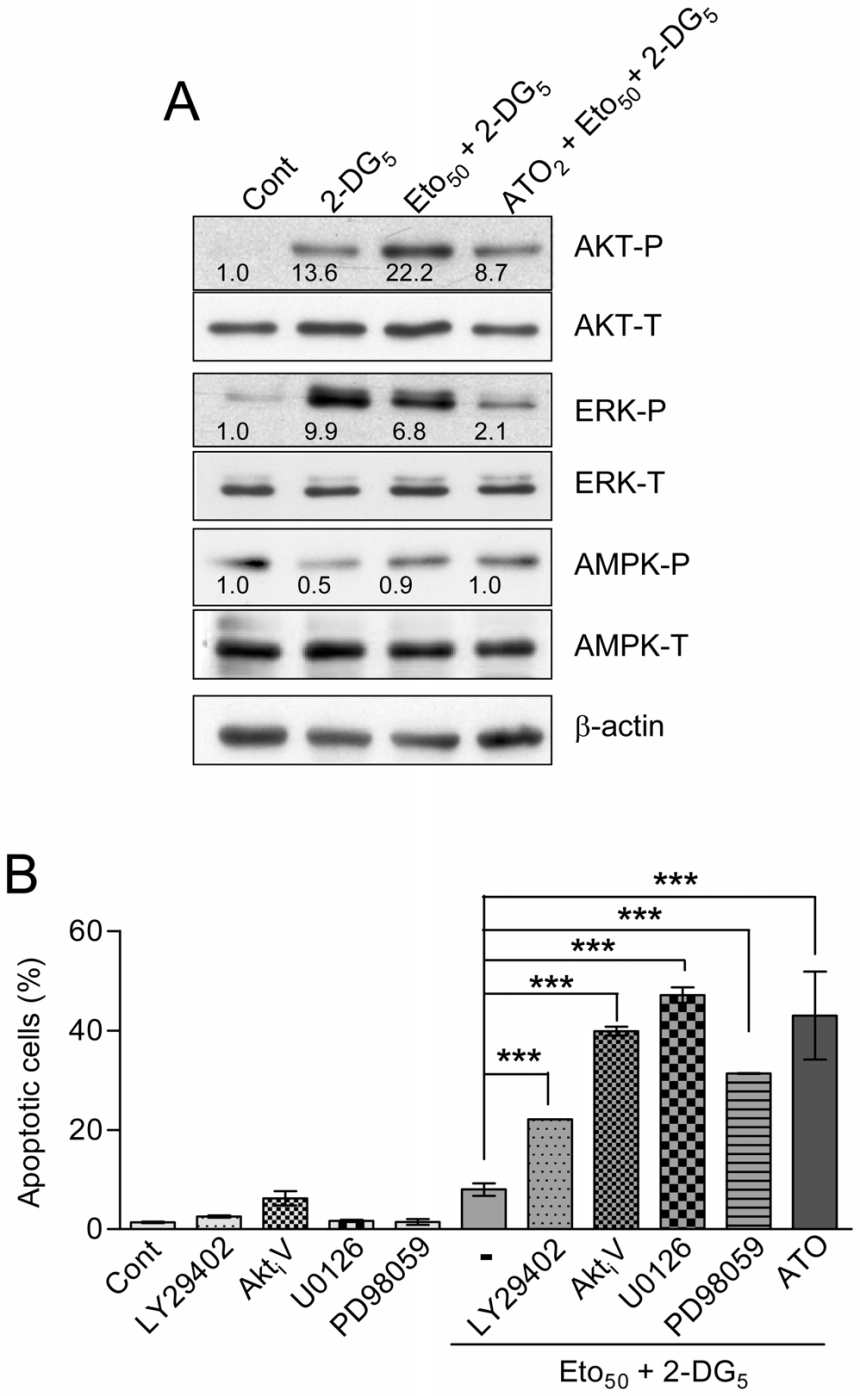
Effect of 2-DG, etomoxir and ATO on protein kinase activities, and effect of protein kinase inhibitors, in HL60 cells. (A) Relative levels of phosphorylated (P) and total (T) Akt, ERKs and AMPK, upon treatment for 4 h with 5 mM 2-DG, alone and in combination with 50 µM etomoxir or etomoxir plus 2 µM ATO. The level of β-actin was assessed as control of sample loading. The numbers inside the blots indicate the values obtained by quantification of band intensities, once normalized in relation to the corresponding β-actin level, and expressed in relation to the Control (arbitrary value of one). The blots are representative of one out of three independent experiments with qualitatively similar results. (B) Frequency of apoptotic cells upon 24 h incubation with etomoxir plus 2-DG, and with the PI3K inhibitor LY 294002 (30 µM), the Akt inhibitor triciribine (Akt_i_V, 10 µM), and the MEK/ERK inhibitors U0126 (5 µM) and PD 98059 (30 µM, alone and in combination with etomoxir plus 2-DG. The triple ATO plus etomoxir plus 2-DG combination is included for comparison. The values represent the mean ± S.D. of four determinations. Symbols indicate significant differences between the indicated pairs of values. All other conditions were as in Figs. 1 and 5.

Looking for possible factors which could explain the increased apoptotic efficacy and sensitizing capacity of etomoxir plus 2-DG, we examined again energy metabolism, oxidative stress, and protein kinase activity modulation in HL60 cells. The results were as follows: (i) Determinations of net ATP levels by bioluminescence assay indicated an approximately 50% decrease upon treatment with 5–10 mM 2-DG alone, which was not significantly augmented by co-treatment with etomoxir or with etomoxir plus ATO (S3B Figure). Determination of adenine nucleotide levels revealed that 2-DG caused a slight decrease in the relative ATP fraction with concomitant increase in the relative ADP and AMP fractions, and a considerable increase in AMP/ATP ratio with concomitant drop in energy charge. This response was particularly evident at 6 h of treatment, and was augmented by co-treatment with etomoxir (etomoxir plus 2-DG), but was not further increased by co-incubation with ATO (ATO plus etomoxir plus 2-DG) (Fig. 7A, B, and S1 Table). (ii) Concerning oxidative stress, 2-DG did not potentiated ROS production, but instead tended to decrease the basal ROS content when used alone, and abrogated the increase caused by etomoxir in the combined treatment. This response was not modified by the inclusion of ATO (Fig. 7C). In addition, the intracellular GSH content was minimally affected by etomoxir plus 2-DG, with or without ATO (Fig. 7D). (iv) Finally, treatment with 2-DG alone greatly stimulated Akt and ERK phosphorylation, and the response was similar (ERK) or even higher (Akt) when combined with etomoxir (Fig. 8A). Of note, AMPK phosphorylation was attenuated by 2-DG alone, as expected [22], but kinase phosphorylation was not increased by etomoxir plus 2-DG, in spite of the increase in AMP/ATP ratio. Importantly, co-incubation with ATO did not affect AMPK phosphorylation, but attenuated slightly (Akt) or strongly (ERK) the increase provoked by etomoxir plus 2-DG (Fig. 8A).

As indicated above, Akt and ERK are normally considered as pro-survival kinases. Because of this, we wanted to know whether the increase in apoptosis obtained by addition of ATO could be mimicked by application of standard kinase inhibitors, namely the phosphatidylinositol 3-kinase (PI3K) inhibitor LY 294002 (30 µM), the Akt inhibitor Akt_i_V (10 µM), and the mitogen-induced extracellular kinase (MEK)/ERK inhibitors U0126 (2.5 µM) and PD 98059 (30 µM). At these concentrations, the lethality of the inhibitors used alone was negligible. It was found that co-incubation with kinase inhibitors greatly stimulated apoptosis generation by etomoxir plus 2-DG (Fig. 8B and S2F Figure). This corroborates that Akt and ERK play defensive roles, and since ATO is able to attenuate kinase activation, provides a partial explanation for the higher apoptotic efficacy of the triple (etomoxir plus 2-DG plus ATO) combination.

## Discussion

The results in this work indicate that the CPT1 inhibitor etomoxir causes concentration-dependent proliferation inhibition and caspase-dependent apoptosis, and sensitizes to apoptosis induction by conventional anti-tumour agents in HL60 AML cells. The lethality of etomoxir was marginal at concentrations up to 100 µM, but the drug efficaciously cooperated to cause apoptosis with sub-lethal ATO concentrations. This effect was corroborated in NB4 APL cells, while both etomoxir lethality and cooperation with ATO were null or negligible in normal proliferating PBLs, suggesting that the response is somewhat selective for tumour cells. Etomoxir also cooperated with the DNA-targeting agents cisplatin and etoposide in HL60 cells, a result consistent with earlier observations in other cell models [11], but the efficacy of this cooperation was clearly lower than in the case of ATO. Interestingly, Samudio et al. [12] reported that etomoxir potentiated apoptosis induction by the Bcl-2 antagonist ABT-737 but not by the DNA-targeting drug cytarabine in AML cells, and proposed that fatty acid inhibitors could be particularly efficacious when combined with direct activators of the intrinsic apoptotic pathway. This is fully consistent with our results, since ATO is a mitochondriotoxic agent, which directly targets the permeability transition pore [43]. However other explanations are also possible – e.g., differential sensitivity of anti-tumour drugs to pro-oxidant environment, as discussed below. We did not directly examine the effect of etomoxir on fatty acid metabolism in HL60 cells. Nonetheless, earlier studies indicated that 50–100 µM etomoxir efficaciously inhibited fatty acid oxidation in other leukemic cell models [14], and we could observe that ATO toxicity was also potentiated by the fatty acid synthesis inhibitor orlistat in HL60 cells (S4A, B Figure). While this might indicate that the chemo-sensitizing action is a consequence of fatty acid oxidation disruption itself, we also observed discrepant effects on cell cycle progression, namely cell accumulation at S phase by etomoxir (Fig. 1B) and at G_1_ by orlistat (S4C, D Figure). Hence, the possible contribution of drug-specific side effects may not be discarded (see below).

Looking for possible factors which could explain the pro-apoptotic action of etomoxir, we firstly examined the capacity to disrupt energy metabolism. Etomoxir was earlier reported to cause ATP depletion in different cell systems, but this effect was generally documented at relatively high drug concentrations (250 µM and above [15]–[17]). In our experiments treatment of HL60 cells with 25–200 µM etomoxir inhibited mitochondrial respiration, with concomitant slight increase in lactate formation. This could represent an adaptive mechanism of the glycolytic machinery to sustain ATP production, and actually the net intracellular ATP content as well as the relative distribution of nucleotide (ATP, ADP and AMP) pools was in general little affected. The observed drastic inhibition of the basal respiration (approximately 65% decrease at 100 µM etomoxir) deserves an additional comment. Since experiments are performed with cells incubated in RPMI containing glucose and glutamine, it is striking that blocking fatty acid oxidation through the inhibition of CPT1 cannot be substituted, at the mitochondrial level, by pyruvate or glutamine oxidation. This would suggest that etomoxir could also be inhibiting electron transport at the respiratory chain. Similar conclusions have been drawn from the analysis of the respiratory capacity of glioblastoma cells treated with etomoxir [17].

As a second parameter we examined oxidative stress, measured by ROS over-accumulation and GSH depletion. As in the case of ATP, earlier reports indicated induction of oxidative stress responses by high concentrations of etomoxir [15]–[17], but another study did not detect ROS induction by low drug concentrations in AML cells [12]. In our experiments 50–200 µM etomoxir caused concentration-dependent, moderate increase in ROS accumulation, and slight decrease in intracellular GSH content, in HL60 cells. The augment in ROS would be surprising if the sole effect of etomoxir is CPT1 inhibition since this would lead to decreased electron flow in the respiratory chain, and hence decreased ROS production by mitochondria. However, if etomoxir is also acting on the respiratory chain, the increased ROS formation would be the expected result of the inhibition of electron flow. In addition, it has been reported that saturated fatty acids such as palmitate stimulate ROS production via activation of NAD(P)H oxidases, and this effect could be mimicked by etomoxir, which contains a saturated fatty acid-derived structure [35], [44]. Unfortunately antioxidants such as N-acetyl-L-cysteine or catalase-polyethylene glycol, satisfactorily used in preceding studies [21], [28], were toxic in combination with etomoxir (data not shown), impeding us to directly prove the functional role of ROS over-accumulation. Nonetheless, we previously demonstrated that AML cells readily tolerate moderate ROS increase and/or GSH depletion [23], [45], so that the here observed fluctuations may not suffice to explain the lethality of etomoxir used alone. By contrast, moderate oxidative stress may account at least in part for the potentiation by etomoxir of ATO-provoked apoptosis, since ATO is an oxidative stress-sensitive drug, the toxicity of which is greatly augmented under conditions of inherent or experimentally-induced ROS increase [28], [37], [45], [46] or GSH depletion [23], [24], [36]. Of note, this is not the case of cisplatin and etoposide, which at low concentrations are little affected by ROS over-production or GSH depletion in AML cells ([23], [28] and data now shown). Hence, taken together these results could explain the differential sensitizing capacity of etomoxir when combined with ATO or with the DNA-targeting drugs.

In addition, we examined the possible role of specific protein kinases on the pro-apoptotic action of etomoxir. Our preceding studies indicated that 2-DG and 3-BrP rapidly stimulated defensive Akt and ERK phosphorylation/activation, but down-regulated LKB-1/AMPK phosphorylation in HL60 cells [22], [23]. This response was apparently surprising, since 2-DG increases the AMP/ATP ratio, which is a trigger of AMPK activation [38], but was adequately explained by the antagonism between AMPK and Akt kinases – i.e., Akt activation is a determinant of AMPK inactivation [22], [23]. In the present experiments etomoxir exerted negligible effect on Akt phosphorylation, and hence there was not impediment for LKB-1/AMPK activation. Nonetheless, the mechanism responsible for this activation is unclear: in fact etomoxir weakly increased the AMP/ATP ratio, but also stimulated ROS production, and AMPK has been characterized as a ROS-inducible kinase [45], [47]. This later possibility could not be directly examined, due to the above indicated toxicity of antioxidant agents. Whatever the case, experiments with the kinase inhibitor compound C revealed that AMPK activation positively regulates apoptosis, and hence could in part account for the potentiation of anti-tumour drug (ATO, cisplatin) lethality in the combined treatments.

Finally, our results indicate that etomoxir efficaciously cooperates with the glycolytic inhibitors 2-DG and lonidamine to cause apoptosis in HL60 cells. This is in agreement with earlier reports using etomoxir plus 2-DG in colon carcinoma cells [11], and is congruent with the concept that glycolysis and fatty acid metabolism are potentially compensatory pathways. This is important, since the clinical efficacy of glycolytic [4], [5] and fatty acid [48] inhibitors in monotherapy is hampered by their low bio-availability and/or side-toxicities at elevated concentrations. Nonetheless, the potential application of this combined treatment exhibits some limitations. Firstly, the efficacy of cooperation between energy pathway inhibitors seems to be conditioned by the inherent metabolic properties of the cancer cell model, as indicated by the poor results obtained with etomoxir plus 2-DG in the highly “glycolytic” NB4 cell line [42]. In addition, etomoxir plus 2-DG also cooperated to induce apoptosis in mitogen-stimulated non-tumour PBLs (although with much lower efficacy than in HL60 cells). Finally, the mechanisms explaining apoptosis potentiation by etomoxir plus 2-DG in HL60 cells are presently unclear. For instance, the combined treatment did not augment oxidative stress, but instead 2-DG abrogated the etomoxir-stimulated ROS production, which is in agreement with earlier reports [49], [50]. In addition, the combined treatment was unable to prevent the activation of defensive Akt and ERK kinases, in comparison to the effect produced by 2-DG alone. Considering energy parameters, incubation with etomoxir plus 2-DG caused an approximately 50% decrease in total net ATP content, but this is similar to the decrease produced by 2-DG alone, and at the lethality of 2-DG at the here-used concentrations (2–10 mM) is negligible. As the most suggestive result, the combination of etomoxir plus 2-DG potentiated the relative reduction in energy charge with a concomitant increase in AMP/ATP ratio, in comparison to either drug alone. Nonetheless, the functional relevance of this alteration in terms of cell viability remains to be determined. As a final step, we observed that the apoptotic efficacy of etomoxir plus 2-DG was further augmented by co-incubation with 2 µM ATO. This may be also relevant in practical terms, since 2 µM ATO is a clinically attainable concentration. Again, this response may not be explained by changes in total ATP content, energy charge, and oxidative stress (ROS production, GSH depletion), since the effects of etomoxir plus 2-DG were not significantly modified by co-incubation with ATO. However, the increase in apoptosis may be at least in part explained by the capacity of ATO to abrogate (ERK) or attenuate (Akt) defensive kinase activation, as indicated by immunoblot assays and corroborated using kinase pharmacologic inhibitors.

**Figure 9:**
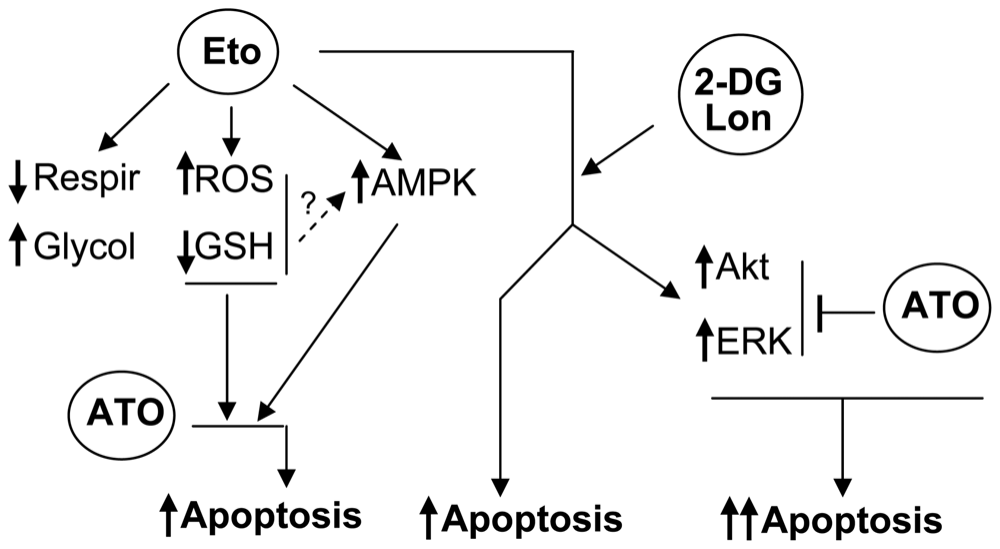
Scheme summarizing the main results in this work. Etomoxir decreases respiration while increasing glycolytic activity. In addition, it causes oxidative stress (ROS over-production, GSH depletion) which, together with AMPK activation, may explain the potentiation of ATO-provoked apoptosis. Etomoxir may also cooperate in some cell models with glycolytic inhibitors (2-DG, lonidamine) to cause apoptosis. This response is further enhanced by co-incubation with ATO, due to the capacity of this agent to attenuate the 2-DG/etomoxir-provoked Akt and ERK activation.

A scheme summarizing the main observations in this work is presented in Fig. 9. Etomoxir causes cyto-reduction (cell cycle disruption, apoptosis induction), and what is more important potentiates the lethality of ATO, and with lower efficacy of other conventional anti-tumour drugs in AML cells. The sensitizing action of etomoxir is not adequately explained by energy depletion, but may be in part explained by the capacity to generate moderate oxidative stress, as evidenced by ROS production and GSH depletion, and to activate the LKB-1/AMPK pro-apoptotic pathway. Moreover, higher apoptotic and sensitizing responses may still be obtained by simultaneously targeting fatty acid and glycolytic metabolism, although this response depends on the used cell type, and the biochemical mechanisms accounting for this enhanced apoptotic response remain to be elucidated. While this is an in vitro pre-clinical assay, we believe that this information might be useful to design new therapeutic approaches based on the use of energy pathway inhibitors.

## Supporting Information

S1 Figure **AMP, ADP and ATP, as determined by HPLC.** The figure shows representative chromatograms identifying adenine nucleotide peaks in (A) untreated HL60 cells (Cont), (B) cells incubated for 6 h with 100 µM etomoxir plus 5 mM 2-DG, and (C) cells incubated for 2 h with 1 µM oligomycin plus 30 mM 2-DG.
(TIF)

S2 Figure **Apoptosis induction, as determined by changes in nuclear morphology.** (A) Examples of chromatin distribution in untreated (Cont) and etomoxir plus ATO-treated HL60 cells. Arrows indicate cells with condensed/fragmented chromatin, and partial chromatin loss, characteristic of late apoptosis. (B–F) Frequency of apoptosis in HL60 cell cultures treated with: (B) the indicated concentrations of etomoxir and ATO, alone and in combination, and with or without z-VAD-fmk; (C) the indicated concentrations of etomoxir and 2-DG, alone and in combination; (D) ATO plus etomoxir plus 2-DG, in relation to ATO plus etomoxir or ATO plus 2-DG; (E) etomoxir plus ATO or cisplatin, in the absence (−) or the presence (+) of the AMPK inhibitor compound C (CC); (F) etomoxir plus 2-DG, in the absence (−) or the presence of the MEK/ERK inhibitor U0126 or the Akt inhibitor Akt_i_V. All treatments lasted for 24 h. ATO was always used at 2 µM. The results represent the mean ± S.D. of at least three determinations. Symbols mean: (*) significant differences between the indicated pairs of values, and (^#^) significant differences between the combined treatment and the sum of values in the corresponding single treatments. For more detailed explanations, see legends of Figures 1 and 6 in the main text.
(TIF)

S3 Figure **Changes in total intracellular ATP levels in HL60 cells.** The bar charts show the changes in ATP, as determined by bioluminescence assays, upon treatment for 4 h with: (A) the indicated concentrations of etomoxir; and (B) the indicated concentrations of etomoxir, 2-DG, and 2 µM ATO, alone and in combination. The results are expressed in relation to the control (approximate ATP content, 20 nmol/10^6^ cells). The results represent the mean ± S.D. of at least four determinations. Symbols indicate significant differences in relation to the control (A), or between the indicated pairs of values (B) (n.s., non-significant). For other conditions, see legend of Figure 1 in the main text.
(TIF)

S4 Figure **Effect of orlistat and ATO on cell viability, apoptosis and cycle phase distribution in HL60 cells.** Cell cultures were incubated for 24 h with the indicated concentrations of orlistat (Orl) and 2 µM ATO, alone and in combination. (A) Changes in the number of viable cells, as evidenced by MTT assay. Absorption values are indicated in relation to untreated (Cont) cultures. (B) Frequency of apoptotic cells, as determined by flow cytometry. (C) Frequency of cells at the different phases of the growth cycle, namely G_1_, S and G_2_/M, and with sub-G_1_ DNA content (apoptotic). Examples of flow cytometry histograms are presented in (D). The results in panels A–C represent the mean ± S.D. of four determinations. Symbols mean: (*) significant differences between the indicated pairs of values, and (^#^) significant differences between the combined treatment and the sum of values in the corresponding single treatments (n.s., non-significant). For more detailed explanations see legend of Figure 1 in the main text.
(TIF)

S1 Table **Effects of 2-DG, etomoxir and ATO on adenine nucleotide pool distribution in HL60 cells.** The table shows the changes in ATP, ADP, AMP pool distribution in untreated cells (Cont), and cells exposed for 2 and/or 6 h to 1 µM oligomycin plus 30 mM 2-DG (positive control), 2-DG (mM), etomoxir (µM) and ATO (µM), alone or in combination. n, number of determinations. Energy charge is defined as: ([ATP]+0.5[ADP])/[ATP+ADP+AMP] (see Atkinson DE (1968) Biochemistry 7: 4030–4034).
(TIF)

## References

[1] Kroemer G, Pouyssegur J (2008) Tumor cell metabolism: cancer's Achilles' heel. Cancer Cell 13:472–482. 18538731 10.1016/j.ccr.2008.05.005

[2] Tennant DA, Duran RV, Gottlieb E (2010) Targeting metabolic transformation for cancer therapy. Nat Rev Cancer 10:267–277. 20300106 10.1038/nrc2817

[3] Zhao Y, Butler EB, Tan M (2013) Targeting cellular metabolism to improve cancer therapeutics. Cell Death Dis 4:e532 10.1038/cddis.2013.60 23470539 PMC3613838

[4] Di Cosimo S, Ferretti G, Papaldo P, Carlini P, Fabi A, et al (2003) Lonidamine: efficacy and safety in clinical trials for the treatment of solid tumors. Drugs Today 39:157–174. 12730701 10.1358/dot.2003.39.3.799451

[5] Dwarakanath B, Jain V (2009) Targeting glucose metabolism with 2-deoxy-D-glucose for improving cancer therapy. Future Oncol 5:581–585. 19519197 10.2217/fon.09.44

[6] Ko YH, Verhoeven HA, Lee MJ, Corbin DJ, Vogl TJ, et al (2012) A translational study "case report" on the small molecule "energy blocker" 3-bromopyruvate (3BP) as a potent anticancer agent: from bench side to bedside. J Bioenerg Biomembr 44:163–170. 22328020 10.1007/s10863-012-9417-4

[7] Harper ME, Antoniou A, Villalobos-Menuey E, Russo A, Trauger R, et al (2002) Characterization of a novel metabolic strategy used by drug-resistant tumor cells. FASEB J 16:1550–1557. 12374777 10.1096/fj.02-0541com

[8] Abdel-Aleem S, Li X, Anstadt MP, Perez-Tamayo RA, Lowe JE (1994) Regulation of glucose utilization during the inhibition of fatty acid oxidation in rat myocytes. Horm Metab Res 26:88–91. 8200620 10.1055/s-2007-1000779

[9] Hubinger A, Knode O, Susanto F, Reinauer H, Gries FA (1997) Effects of the carnitine-acyltransferase inhibitor etomoxir on insulin sensitivity, energy expenditure and substrate oxidation in NIDDM. Horm Metab Res 29:436–439. 9370111 10.1055/s-2007-979072

[10] Abozguia K, Clarke K, Lee L, Frenneaux M (2006) Modification of myocardial substrate use as a therapy for heart failure. Nat Clin Pract Cardiovasc Med 3:490–498. 16932766 10.1038/ncpcardio0583

[11] Hernlund E, Ihrlund LS, Khan O, Ates YO, Linder S, et al (2008) Potentiation of chemotherapeutic drugs by energy metabolism inhibitors 2-deoxyglucose and etomoxir. Int J Cancer 123:476–483. 18452174 10.1002/ijc.23525

[12] Samudio I, Harmancey R, Fiegl M, Kantarjian H, Konopleva M, et al (2010) Pharmacologic inhibition of fatty acid oxidation sensitizes human leukemia cells to apoptosis induction. J Clin Invest 120:142–156. 20038799 10.1172/JCI38942 PMC2799198

[13] Li J, Zhao S, Zhou X, Zhang T, Zhao L, et al (2013) Inhibition of lipolysis by mercaptoacetate and etomoxir specifically sensitize drug-resistant lung adenocarcinoma cell to paclitaxel. PLoS ONE 8:e74623 10.1371/journal.pone.0074623 24040298 PMC3770579

[14] Tirado-Vélez JM, Joumady I, Sáez-Benito A, Cózar-Castellano I, Perdomo G (2012) Inhibition of fatty acid metabolism reduces human myeloma cells proliferation. PLoS ONE 7:e46484 10.1371/journal.pone.0046484 23029529 PMC3460894

[15] Merrill CL, Ni H, Yoon LW, Tirmenstein MA, Narayanan P, et al (2002) Etomoxir-induced oxidative stress in HepG2 cells detected by differential gene expression is confirmed biochemically. Toxicol Sci 68:93–101. 12075114 10.1093/toxsci/68.1.93

[16] Vickers AE, Bentley P, Fisher RL (2006) Consequences of mitochondrial injury induced by pharmaceutical fatty acid oxidation inhibitors is characterized in human and rat liver slices. Toxicol In Vitro 20:1173–1182. 16545538 10.1016/j.tiv.2006.01.021

[17] Pike LS, Smift AL, Croteau NJ, Ferrick DA, Wu M (2011) Inhibition of fatty acid oxidation by etomoxir impairs NADPH production and increases reactive oxygen species resulting in ATP depletion and cell death in human glioblastoma cells. Biochim Biophys Acta 6:726–734. 10.1016/j.bbabio.2010.10.022 21692241

[18] Breccia M, Lo-Coco F (2012) Arsenic trioxide for management of acute promyelocytic leukemia: current evidence on its role in front-line therapy and recurrent disease. Expert Opin Pharmacother 13:1031–1043. 22468778 10.1517/14656566.2012.677436

[19] Amadori S, Fenaux P, Ludwig H, O'Dwyer M, Sanz M (2005) Use of arsenic trioxide in haematological malignancies: insight into the clinical development of a novel agent. Curr Med Res Opin 21:403–41. 15811209 10.1185/030079904X20349

[20] Kritharis A, Bradley TP, Budman DR (2013) The evolving use of arsenic in pharmacotherapy of malignant disease. Ann Hematol 92:719–730. 23494203 10.1007/s00277-013-1707-3

[21] Calviño E, Estañ MC, Simón GP, Sancho P, Boyano-Adánez MC, et al (2011) Increased apoptotic efficacy of lonidamine plus arsenic trioxide combination in human leukemia cells. Reactive oxygen species generation and defensive protein kinase (MEK/ERK, Akt/mTOR) modulation. Biochem Pharmacol 82:1619–1629. 21889928 10.1016/j.bcp.2011.08.017

[22] Estañ MC, Calviño E, De Blas E, Boyano-Adánez MC, Mena ML, et al (2012) 2-Deoxy-D-glucose cooperates with arsenic trioxide to induce apoptosis in leukemia cells: involvement of IGF-1R-regulated Akt/mTOR, MEK/ERK and LKB-1/AMPK signaling pathways. Biochem Pharmacol 84:1604–1616. 23041229 10.1016/j.bcp.2012.09.022

[23] Calviño E, Estañ MC, Sánchez-Martín C, Brea R, De Blas E, et al (2014) Regulation of death induction and chemosensitizing action of 3-bromopyruvate in myeloid leukemia cells: energy depletion, oxidative stress, and protein kinase activity modulation. J Pharmacol Exp Ther 348:324–335. 24307199 10.1124/jpet.113.206714

[24] Dai J, Weinberg RS, Waxman S, Jing Y (1999) Malignant cells can be sensitized to undergo growth inhibition and apoptosis by arsenic trioxide through modulation of the glutathione redox system. Blood 93:268–277. 9864170

[25] Collins SJ, Gallo RC, Gallagher RE (1977) Continuous growth and differentiation of human myeloid leukaemic cells in suspension culture. Nature 270:347–349. 271272 10.1038/270347a0

[26] Lanotte M, Martin-Thouvenin V, Najman S, Balerini P, Valensi F, et al (1991) NB4, a maturation inducible cell line with t(15;17) marker isolated from a human acute promyelocytic leukemia (M3). Blood 77:1080–1086. 1995093

[27] Troyano A, Fernández C, Sancho P, De Blas E, Aller P (2001) Effect of glutathione depletion on antitumor drug toxicity (apoptosis and necrosis) in U-937 human promonocytic cells. The role of intracellular oxidation. J Biol Chem 50:47107–47115. 10.1074/jbc.M104516200 11602574

[28] Sánchez Y, Simón GP, Calviño E, De Blas E, Aller P (2010) Curcumin stimulates reactive oxygen species production and potentiates apoptosis induction by the antitumor drugs arsenic trioxide and lonidamine in human myeloid leukemia cell lines. J Pharmacol Exp Ther 335:114–123. 20605902 10.1124/jpet.110.168344

[29] De Korte D, Haverkort WA, Van Gennip AH, Roos D (1985) Nucleotide profiles of normal human blood cells determined by high-performance liquid chromatography. Anal Biochem 147:197–209. 4025817 10.1016/0003-2697(85)90028-4

[30] Galán A, García-Bermejo ML, Troyano A, Vilaboa NE, De Blas E, et al (2000) Stimulation of p38 mitogen-activated protein kinase is an early regulatory event for the cadmium-induced apoptosis in human promonocytic cells. J Biol Chem 275:11418–11424. 10753958 10.1074/jbc.275.15.11418

[31] Chou TC, Talalay P (1984) Quantitative analysis of dose-effect relationships: the combined effects of multiple drugs or enzyme inhibitors. Adv Enzyme Regul 22:27–55. 6382953 10.1016/0065-2571(84)90007-4

[32] Miller WH Jr, Schipper HM, Lee JS, Singer J, Waxman S (2002) Mechanisms of action of arsenic trioxide. Cancer Res 62:3893–3903. 12124315

[33] Lieberthal W, Menza SA, Levine JS (1998) Graded ATP depletion can cause necrosis or apoptosis of cultured mouse proximal tubular cells. Am J Physiol 274:F315–327. 9486226 10.1152/ajprenal.1998.274.2.F315

[34] Izyumov DS, Avetisyan AV, Pletjushkina OY, Sakharov DV, Wirtz KW, et al (2004) "Wages of fear": transient threefold decrease in intracellular ATP level imposes apoptosis. Biochim Biophys Acta 23:141–147. 10.1016/j.bbabio.2004.05.007 15282185

[35] Cabrero A, Alegret M, Sánchez RM, Adzet T, Laguna JC, et al (2002) Increased reactive oxygen species production down-regulates peroxisome proliferator-activated alpha pathway in C2C12 skeletal muscle cells. J Biol Chem 277:10100–10107. 11792699 10.1074/jbc.M110321200

[36] Yang CH, Kuo ML, Chen JC, Chen YC (1999) Arsenic trioxide sensitivity is associated with low level of glutathione in cancer cells. Br J Cancer 81:796–799. 10555748 10.1038/sj.bjc.6690766 PMC2374294

[37] Yi J, Gao F, Shi G, Li H, Wang Z, et al (2002) The inherent cellular level of reactive oxygen species: one of the mechanisms determining apoptotic susceptibility of leukemic cells to arsenic trioxide. Apoptosis 7:209–215. 11997664 10.1023/a:1015331229263

[38] Hardie DG (2007) AMP-activated protein kinase as a drug target. Annu Rev Pharmacol Toxicol 7:185–210. 10.1146/annurev.pharmtox.47.120505.105304 16879084

[39] Kuznetsov JN, Leclerc GJ, Leclerc GM, Barredo JC (2011) AMPK and Akt determine apoptotic cell death following perturbations of one-carbon metabolism by regulating ER stress in acute lymphoblastic leukemia. Mol Cancer Ther 10:437–447. 21262957 10.1158/1535-7163.MCT-10-0777 PMC3053424

[40] Tsunekawa-Imai N, Miwa H, Shikami M, Suganuma K, Goto M, et al (2013) Growth of xenotransplanted leukemia cells is influenced by diet nutrients and is attenuated with 2-deoxyglucose. Leuk Res 37:1132–1136. 23806233 10.1016/j.leukres.2013.05.017

[41] Scotland S, Saland E, Skuli N, De Toni F, Boutzen H, et al (2013) Mitochondrial energetic and AKT status mediate metabolic effects and apoptosis of metformin in human leukemic cells. Leukemia 27:2129–2138. 23568147 10.1038/leu.2013.107 PMC10869165

[42] Suganuma K, Miwa H, Imai N, Shikami M, Goto M, et al (2010) Energy metabolism of leukemia cells: glucolysis versus oxidative phosphorylation. Leuk Lymphoma 51:2112–2119. 20860495 10.3109/10428194.2010.512966

[43] Fantin VR, Leder P (2006) Mitochondriotoxic compounds for cancer therapy. Oncogene 25:4787–4797. 16892091 10.1038/sj.onc.1209599

[44] Inoguchi T, Li P, Umeda F, Yu HY, Kakimoto M, et al (2000) High glucose level and free fatty acid stimulate reactive oxygen species production through protein kinase C-dependent activation of NAD(P)H oxidase in cultured vascular cells. Diabetes 49:1939–1945. 11078463 10.2337/diabetes.49.11.1939

[45] Sánchez Y, Amrán D, Fernández C, De Blas E, Aller P (2008) Genistein selectively potentiates arsenic trioxide-induced apoptosis in human leukemia cells via reactive oxygen species generation and activation of reactive oxygen species-inducible protein kinases (p38-MAPK, AMPK). Int J Cancer 123:1205–1214. 18546268 10.1002/ijc.23639

[46] Diaz Z, Colombo M, Mann KK, Su H, Smith KN, et al (2005) Trolox selectively enhances arsenic-mediated oxidative stress and apoptosis in APL and other malignant cell lines. Blood 105:1237–1245. 15466933 10.1182/blood-2004-05-1772

[47] Emerling BM, Weinberg F, Snyder C, Burgess Z, Mutlu GM, et al (2009) Hypoxic activation of AMPK is dependent on mitochondrial ROS but independent of an increase in AMP/ATP ratio. Free Radic Biol Med 46:1386–1391. 19268526 10.1016/j.freeradbiomed.2009.02.019 PMC3326346

[48] Holubarsch CJ, Rohrbach M, Karrasch M. Clin Sci 113:205–212. 17319797 10.1042/CS20060307

[49] Duan W, Mattson MP (1999) Dietary restriction and 2-deoxyglucose administration improve behavioral outcome and reduce degeneration of dopaminergic neurons in models of Parkinson's disease. J Neurosci Res 57:195–206. 10398297 10.1002/(SICI)1097-4547(19990715)57:2<195::AID-JNR5>3.0.CO;2-P

[50] Xi H, Barredo JC, Merchan JR, Lampidis TJ (2013) Endoplasmic reticulum stress induced by 2-deoxyglucose but not glucose starvation activates AMPK through CaMKKKβ leading to autophagy. Biochem Pharmacol 85:1463–1477. 23500541 10.1016/j.bcp.2013.02.037

