# Supplementary figures and images for: Apoptotic Efficacy of Etomoxir in Human Acute Myeloid Leukemia Cells. Cooperation with Arsenic Trioxide and Glycolytic Inhibitors, and Regulation by Oxidative Stress and Protein Kinase Activities
Source: PLoS One. 2014 Dec 15;9(12):e115250. doi: 10.1371/journal.pone.0115250 (PMC4266683; doi:10.1371/journal.pone.0115250)

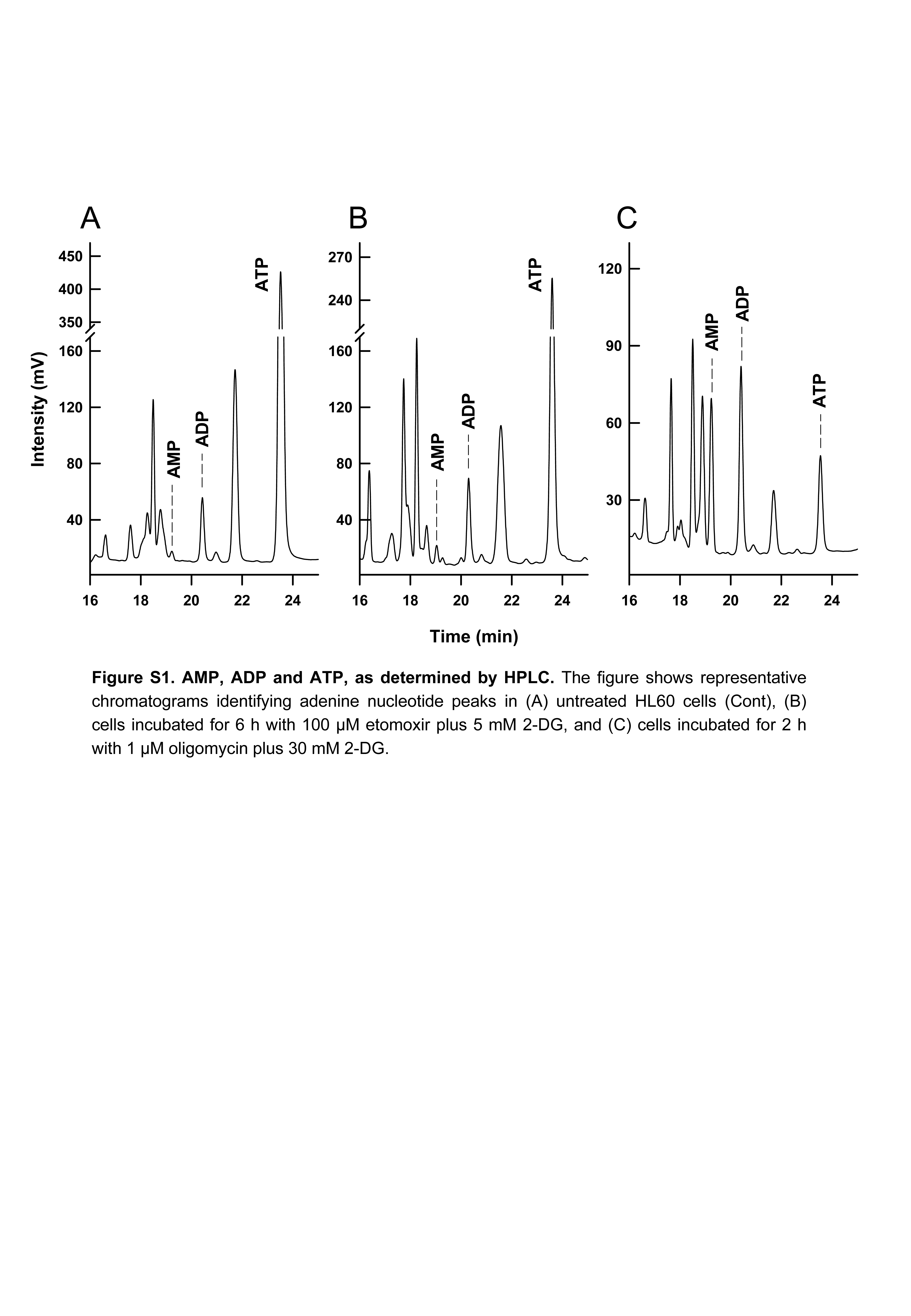

Supplement: S1 Figure — AMP, ADP and ATP, as determined by HPLC. The figure shows representative chromatograms identifying adenine nucleotide peaks in (A) untreated HL60 cells (Cont), (B) cells incubated for 6 h with 100 µM etomoxir plus 5 mM 2-DG, and (C) cells incubated for 2 h with 1 µM oligomycin plus 30 mM 2-DG. (TIF) [file pone.0115250.s001.tif]

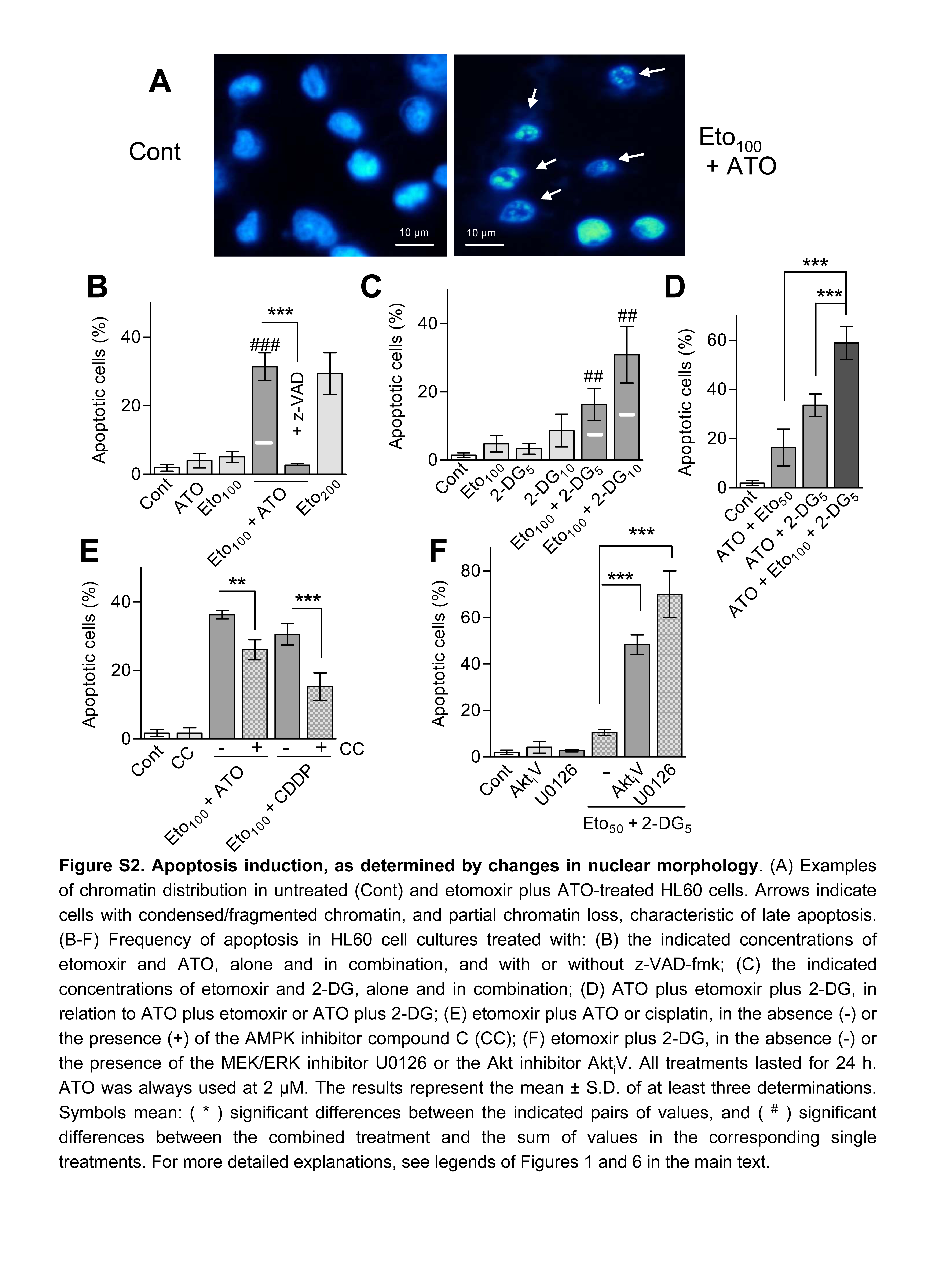

Supplement: S2 Figure — Apoptosis induction, as determined by changes in nuclear morphology. (A) Examples of chromatin distribution in untreated (Cont) and etomoxir plus ATO-treated HL60 cells. Arrows indicate cells with condensed/fragmented chromatin, and partial chromatin loss, characteristic of late apoptosis. (B–F) Frequency of apoptosis in HL60 cell cultures treated with: (B) the indicated concentrations of etomoxir and ATO, alone and in combination, and with or without z-VAD-fmk; (C) the indicated concentrations of etomoxir and 2-DG, alone and in combination; (D) ATO plus etomoxir plus 2-DG, in relation to ATO plus etomoxir or ATO plus 2-DG; (E) etomoxir plus ATO or cisplatin, in the absence (−) or the presence (+) of the AMPK inhibitor compound C (CC); (F) etomoxir plus 2-DG, in the absence (−) or the presence of the MEK/ERK inhibitor U0126 or the Akt inhibitor AktiV. All treatments lasted for 24 h. ATO was always used at 2 µM. The results represent the mean ± S.D. of at least three determinations. Symbols mean: (*) significant differences between the indicated pairs of values, and (#) significant differences between the combined treatment and the sum of values in the corresponding single treatments. For more detailed explanations, see legends of Figures 1 and 6 in the main text. (TIF) [file pone.0115250.s002.tif]

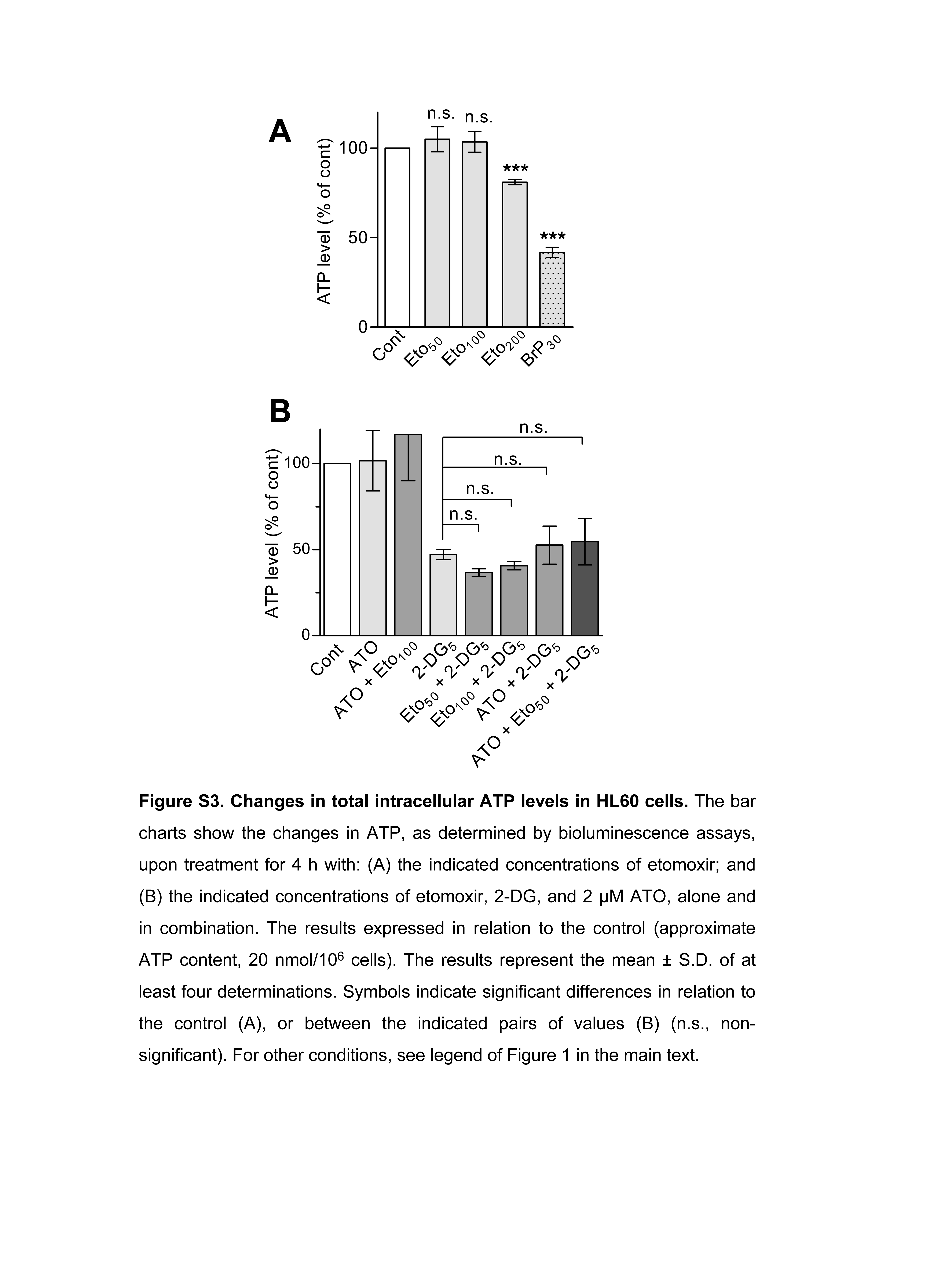

Supplement: S3 Figure — Changes in total intracellular ATP levels in HL60 cells. The bar charts show the changes in ATP, as determined by bioluminescence assays, upon treatment for 4 h with: (A) the indicated concentrations of etomoxir; and (B) the indicated concentrations of etomoxir, 2-DG, and 2 µM ATO, alone and in combination. The results are expressed in relation to the control (approximate ATP content, 20 nmol/106 cells). The results represent the mean ± S.D. of at least four determinations. Symbols indicate significant differences in relation to the control (A), or between the indicated pairs of values (B) (n.s., non-significant). For other conditions, see legend of Figure 1 in the main text. (TIF) [file pone.0115250.s003.tif]

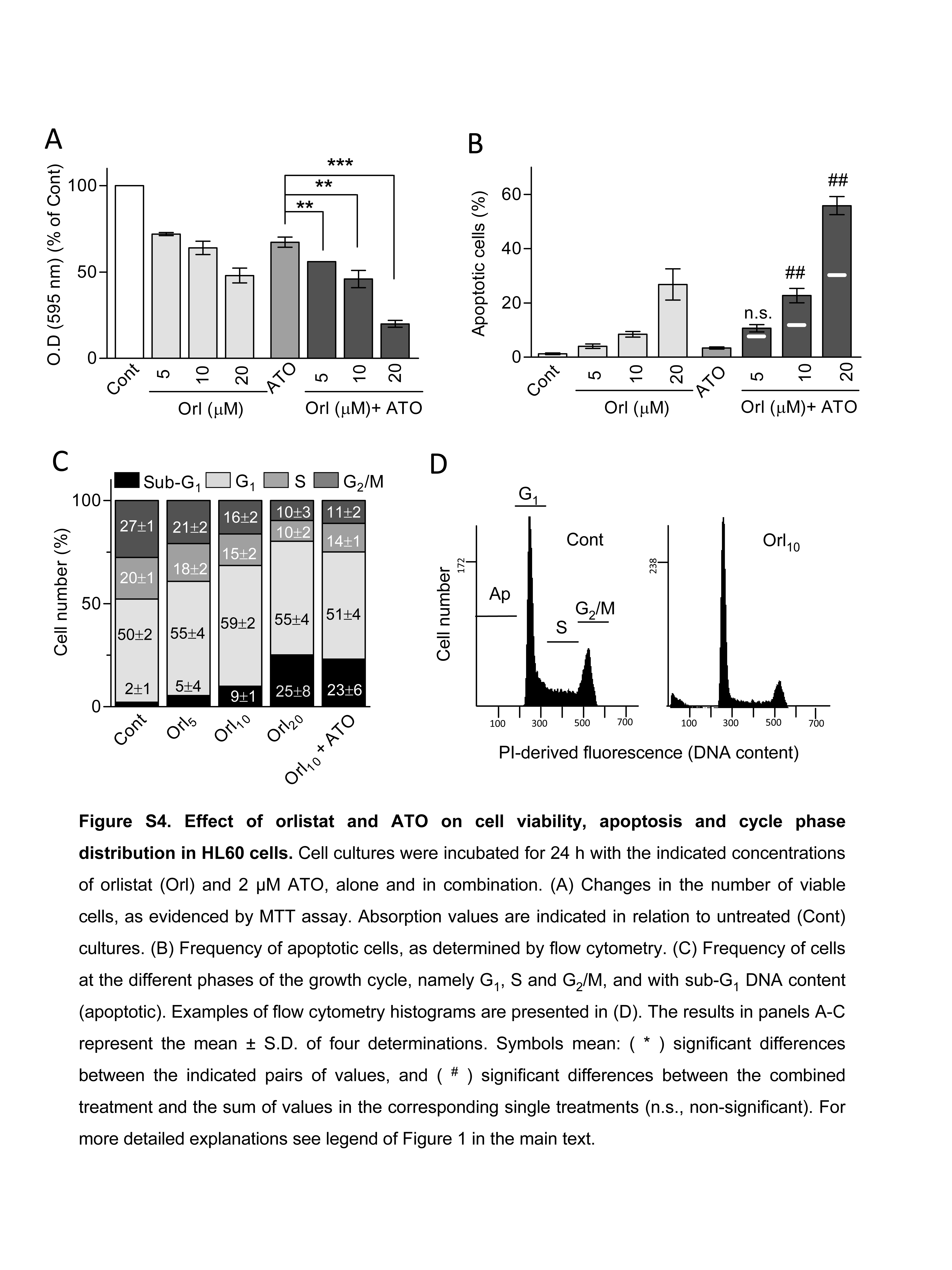

Supplement: S4 Figure — Effect of orlistat and ATO on cell viability, apoptosis and cycle phase distribution in HL60 cells. Cell cultures were incubated for 24 h with the indicated concentrations of orlistat (Orl) and 2 µM ATO, alone and in combination. (A) Changes in the number of viable cells, as evidenced by MTT assay. Absorption values are indicated in relation to untreated (Cont) cultures. (B) Frequency of apoptotic cells, as determined by flow cytometry. (C) Frequency of cells at the different phases of the growth cycle, namely G1, S and G2/M, and with sub-G1 DNA content (apoptotic). Examples of flow cytometry histograms are presented in (D). The results in panels A–C represent the mean ± S.D. of four determinations. Symbols mean: (*) significant differences between the indicated pairs of values, and (#) significant differences between the combined treatment and the sum of values in the corresponding single treatments (n.s., non-significant). For more detailed explanations see legend of Figure 1 in the main text. (TIF) [file pone.0115250.s004.tif]

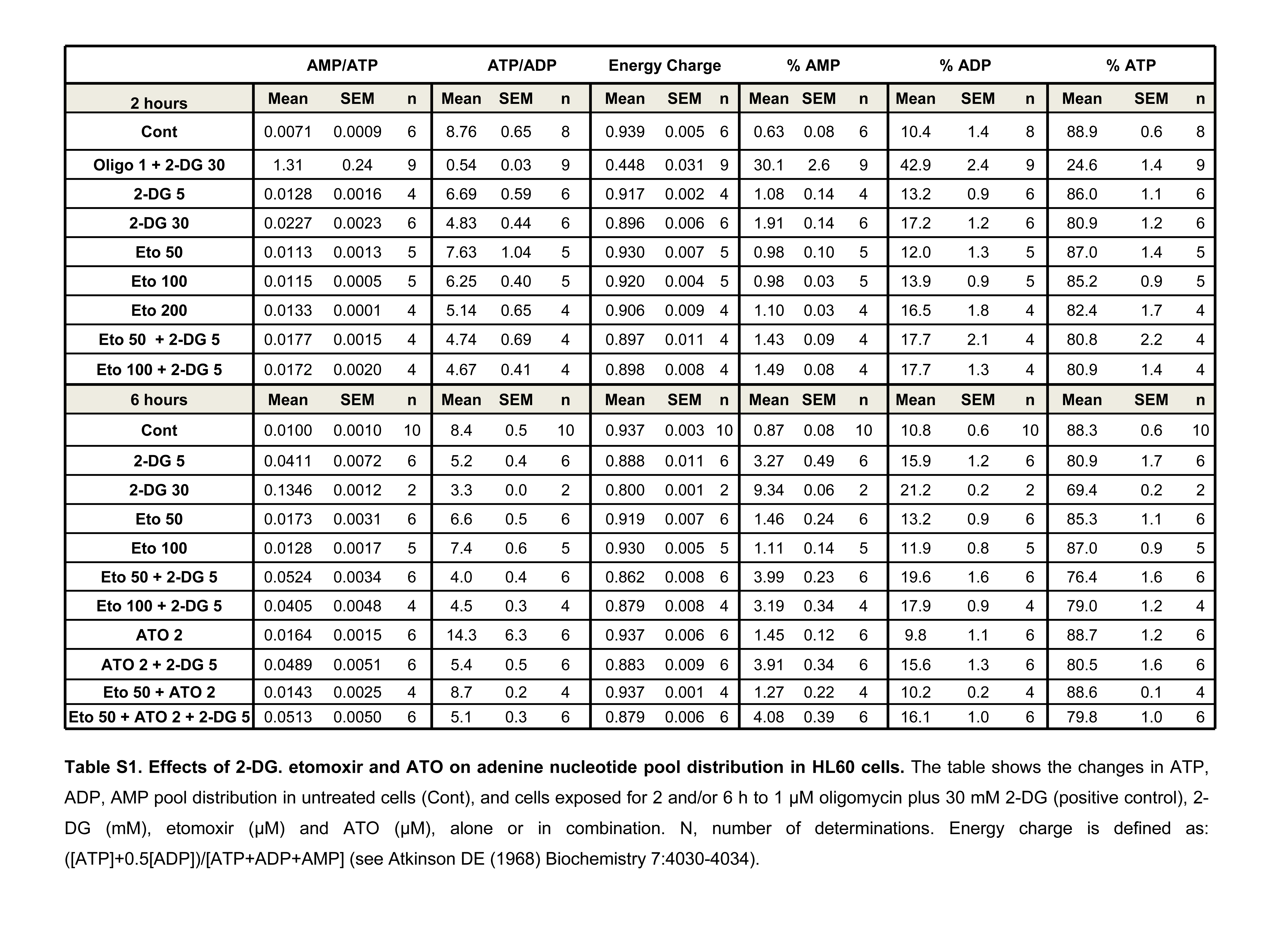

Supplement: S1 Table — Effects of 2-DG, etomoxir and ATO on adenine nucleotide pool distribution in HL60 cells. The table shows the changes in ATP, ADP, AMP pool distribution in untreated cells (Cont), and cells exposed for 2 and/or 6 h to 1 µM oligomycin plus 30 mM 2-DG (positive control), 2-DG (mM), etomoxir (µM) and ATO (µM), alone or in combination. n, number of determinations. Energy charge is defined as: ([ATP]+0.5[ADP])/[ATP+ADP+AMP] (see Atkinson DE (1968) Biochemistry 7: 4030–4034). (TIF) [file pone.0115250.s005.tif]
